# Modelling the Effects of Forest use Change on Brownification of Finnish Rivers under Atmospheric Pressure

**DOI:** 10.1007/s00267-024-02058-1

**Published:** 2024-10-18

**Authors:** Katri Rankinen, Jose E. Cano Bernal, Maria Holmberg, Magnus Nordling, Torsti Schulz, Annikki Mäkelä, Ninni Mikkonen, Heini Kujala, Leah Jackson-Blake, Heleen A. de Wit, Martin Forsius

**Affiliations:** 1 Finnish Environment Institute: Latokartanonkaari 11, 00790 Helsinki, Finland; 2 Norwegian Institute for Water Research: Økernveien 94, 0579 Oslo, Norway; 3 University of Helsinki, P.O. Box 4 (Yliopistonkatu 3), 00014 University of Helsinki, Helsinki, Finland

**Keywords:** Brownification, TOC, Acid deposition, Climate change, Forest use change, Mathematical modelling

## Abstract

Browning of surface waters due to increased terrestrial loading of dissolved organic matter (DOM) is observed across the Northern Hemisphere. The effects influence several ecosystem services from freshwater productivity to water purification. Brownification is often explained by changes in large-scale anthropogenic pressures and ecosystem functioning (acidification, climate change, and land cover changes). This study examined the effect of forest use changes on water browning in Finland, considering the effects of global pressures. Our goal was to find the ecosystems and geographic areas that are most sensitive to environmental pressures that increase the loading of DOM. We were also looking for land use strategies that decrease browning. We combined mathematical watershed modelling to scenarios of climate change, atmospheric deposition, and forest use change. Changes included scenarios of forest harvest and protection on forest, that were derived from European Union’s regulation. The study area covered 20 watersheds from south to north of Finland. In northern Finland brownification continue. In southern Finland global influence (atmospheric deposition, climate change) seem to weaken, giving more space for local forest use change having an influence on brownification. Forest use change was more influential in river basins dominated by organic soils than in mineral soils. Extending forest protection decreased brownification especially in areas where the influence of atmospheric pressure is decreasing. When forest protection is planned to provide a carbon storage and sequestration potential and to favor biodiversity, it has favorable effect on surface water quality as well.

## Introduction

Browning of surface waters due to increased terrestrial loading of dissolved organic matter (DOM) is observed across the Northern Hemisphere. This increase has widely been studied as it may be a considerable part of areal carbon balance (de Wit et al. 2015). The effects of brownification extend to ecosystem services like water purification, but also freshwater productivity through limiting light penetration and creating more stable thermal stratification. Increased DOM is challenging for drinking water treatment, impacts surface water productivity (Lyche Solheim et al. 2024), increases the growth of nuisance algae (Rohrlack 2023) and impacts greenhouse gas production. Browning of surface water also decrease the recreation value of water bodies (Krzeminsk et al. 2019; Kritzberg et al. 2020). Thus, understanding the factors controlling the export of DOM from catchments is important for predictions of future browning and its management (Roulet and Moore 2006).

Brownification is often explained by changes in large-scale anthropogenic pressures and ecosystem functioning, including acidification, climate change, and land cover changes. A decrease in sulfur (SO_4_) deposition is assumed to increase soil organic matter solubility (de Wit et al. 2007; Monteith et al. 2007). Climate change influences brownification by increasing temperatures and thus stimulating the decay of dissolved organic carbon (DOC) in soils, and by changing the timing and intensity of precipitation and snowmelt (Hongve et al. 2004; Erlandsson et al. 2008). Land use or cover changes and forestry measures (Mattsson et al. 2005; Nieminen et al. 2020; Estlander et al. 2021; Nieminen et al. 2021; Skerlep 2021; Williamson et al. 2021; Cano Bernal et al. 2022; Škerlep et al. 2022) have recently been observed to be one reason for increased concentrations and transport of DOC. Combinations of these factors (Erlandsson et al. 2008) may explain many of the observed trends.

There have been considerable changes in these environmental pressures (atmospheric deposition, climate and land cover) during the last decades. Acidic deposition has decreased considerably since 1990’s due to a successful air protection policy. Thus, increased DOC loading is assumed to result from a process of chemical recovery in terrestrial ecosystems. At the same time, climate change has increased air temperatures and changed precipitation patterns. Changing climate is assumed to increase nutrient loading from Nordic river basins due to increase in temperature, precipitation and extreme events (Puustinen et al. 2007; Brookshire et al. 2011; Øygarden et al. 2014). Expected climate change in Finland for the period 2040–2069 based on 30 global climate models, project mean temperatures increase by 2.4 (1.0–3.8) ◦C in summer and 3.3 (1.2–5.4) °C in winter (the multi-model mean change relative to 1981– 2010). Precipitation increases by 5 (−6 to 17) % in summer and 12 (0–24) % in winter (Ruosteenoja and Jylhä 2021).

In Finland, productive forests cover about 66% of the land area (Vaahtera et al. 2021). Around 90% of these forests are coniferous managed forests under even-aged rotation forestry, and the rest is protected. Forests have always been used intensively in Finland (Tasanen 2004) but since the middle of the last century, forest management has largely aimed at increasing wood production. Forestry is mainly based on traditional clear-cutting combined with regeneration measures, like mounding and furrowing. As a result, there has been a significant increase in both volume growth and extraction of timber during the last 100 years. In addition, forest management has altered age-structure and functional heterogeneity of the Finnish forests (Korhonen et al. 2021), reducing the area of old-growth forests (Keto-Tokoi and Kuuluvainen 2014). Notable amounts of naturally open peatlands have been drained to increase forest productivity to allow economic forestry and these areas are kept actively dry with the ditches. In peatland forests, the network of ditches is also actively maintained. Over half of the mires of Finland (approximately 5.7 million ha) have been drained for forestry, mostly since 1950 (Turunen 2008) and additionally about 0.6 million ha of mineral soils have been drained for forestry (Korhonen et al. 2021).

Forest management methods and harvest intensities influence the carbon sequestration and biodiversity of forests, in addition to wood production. Currently, there is a strong focus on protecting biodiversity and mitigating climate change in European environmental policy. Main policy instruments are the EU Green Deal (European Commission 2021a), EU Biodiversity Strategy 2030 (European Commission 2020), and the Regulation for the Land Use, Land Use Change and Forestry (LULUCF) sector (European Commission 2021b). Finland is committed to the EU’s goal of protecting 30% of land and sea areas, and 10% of them strictly (CBD 2022). The LULUCF regulation outlines how carbon sinks and greenhouse gas emissions from the land use sector are considered in the EU’s climate goals until 2030. When studying environmental effects of land cover changes due to these policies, environmental influence on biodiversity, sustainability of forestry, and water quality should be simultaneously considered (Alam et al. 2023; Forsius et al. 2023).

Different mathematical models can be used to study the environmental effects and to optimize best management regimes. Quantitative, process-based models are widely used for assessing the combined effects of climate and /land cover change (e.g. Moe et al. 2019; Whitehead et al. 2013; Bussi et al. 2016; Molina-Navarro et al. 2018) on catchment processes. The results have provided an important source of information to decision-makers for estimating the impact of land management alternatives on water quality. Different examples of these catchment-scale, process-based models have been applied in the Nordic countries to estimate the impact of land use/land cover and land management on water quality (e.g. Granlund et al. 2004; Räsänen et al. 2006; Rankinen et al. 2019; Farkas et al. 2013; Lu et al. 2016), on biodiversity, and on carbon sequestration (Kujala et al. 2023; Mäkelä et al. 2023).

In this study we were looking for land use strategies that decrease browning. The main aim was to answer the following questions: 1. What is the effect of local forest use change in forested land on TOC loading from river basins given global changes of anthropogenic pressures (climate and deposition) in Finland? 2. Where are the most sensitive areas for browning? 3. How are forest use strategies related to other land use strategies? Forest use change included scenarios of forest harvest and protection on forest (Kujala et al. 2023; Mäkelä et al. 2023), that were derived from EU’s Biodiversity Strategy and LULUCF regulation. We did not include the change in land use between forest and other forms of land use (e:g: agricultural land). We combined mathematical watershed scale modelling to scenarios of climate change, atmospheric deposition, and forest use change.

## Material and Methods

### Area

**Fig. 1:**
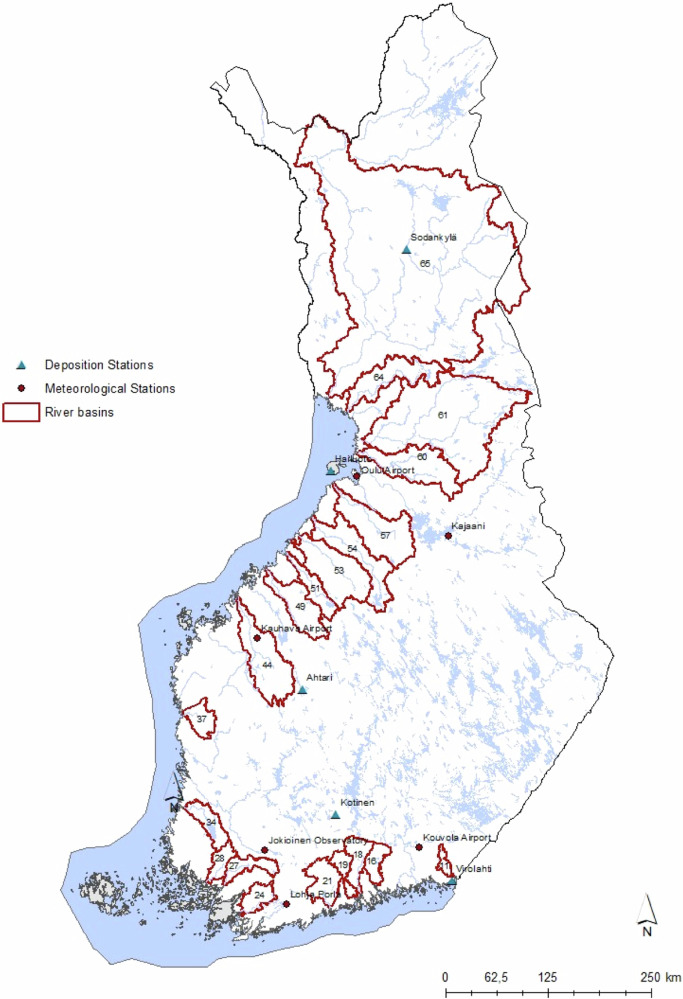
Location of river basins and meteorological and deposition stations. River basin group one is basins 11–21, group two basins 24–37, group three basins 44–64 and group four basin 65

We considered 20 Finnish river basins discharging to the Baltic Sea (Fig. 1). These rivers have long-term monitoring time series of discharge and TOC concentrations at the outlet of the river. The samples of river basins were divided into four smaller groups. This division was based on climatological, geographical and soil type characteristics, in particular mineral and organic soils (Table 1). The southern catchments discharge into the Gulf of Finland, and mineral soil types are dominating. The second group of catchments discharges to the Archipelago Sea, and they are characterized by erosion-prone clay soils and a high proportion of agricultural fields. The third group of river basins discharge into the Bothnian Sea and they are characterized by a high presence of organic soils. Lastly, the northernmost river basin in Lapland, forming the fourth group, is influenced by cold climatic conditions and mineral soils.

**Table 1:** Land use/cover in river basins (based on CORINE2018 and MS-NFI 2019)

Code	River basin group	Name	Area	Agri	ForMin	ForOrg	ForMinCut	ForOrgCut	OldFor	Wetland	Water
			[km^2^]	[%]	[%]	[%]	[%]	[%]	[%]	[%]	[%]
11	1	Virojoki	357	16	58	9	11	1	0	1	3
16	1	Koskenkylänjoki	895	34	47	3	10	0	0.1	0	4
18	1	Porvoonjoki	1273	39	47	2	10	0	0	0	2
19	1	Mustijoki	783	36	46	5	10	1	0.1	0	2
21	1	Vantaanjoki	1686	41	42	5	9	1	0.1	0	2
24	2	Kiskonjoki	629	27	46	5	10	1	0.5	1	6
27	2	Paimionjoki	1088	48	38	3	8	0	0.1	0	2
28	2	Aurajoki	874	46	39	4	8	1	0.3	0	1
34	2	Eurajoki	1336	28	43	5	8	1	0.2	1	13
37	2	Lapväärtinjoki	1098	15	49	18	8	3	0.3	1	0
44	3	Lapuanjoki	4122	26	46	15	7	2	0.3	2	3
49	3	Perhonjoki	2524	14	42	21	6	4	0.2	2	3
51	3	Lestijoki	1373	13	41	21	6	3	0.2	1	6
53	3	Kalajoki	3658	19	47	19	7	2	0.3	1	2
54	3	Pyhäjoki	3712	13	46	21	8	3	0.3	1	5
57	3	Siikajoki	4318	12	36	31	7	6	0.1	1	2
60	3	Kiiminginjoki	3814	3	39	25	6	8	1	1	3
61	3	Iijoki	14179	2	47	20	7	6	4	1	6
64	3	Simojoki	3157	2	40	24	5	6	2	1	6
65	4	Kemijoki	51086	1	52	14	8	4	14	0	5

Code is according to the national classification of river basins (Ekholm. 1993)

*Agri* agricultural area, *ForMin* economical forest on mineral soil, *ForOrg* economical forest on organic soil, *ForMinCut* forest cuttings on mineral soil, *ForOrgCut* forest cuttings on organic soil, *OldFor* over 120 year old forest

Typically, economic forest is felled at the age of 60–80 years. The area of old forest (>120 years) is low in southern Finland, and only in Lapland the area exceeds 10% of the river basin area (Korhonen et al. 2021). Forestry influences the forest structure strongly. In the two southern river basin groups (1 and 2) the highest basal area is in the age class 80–120 years (overaged economic forests), but in northern Finland in the age class over 120 years. In the south, forests exceeding 120 years are mostly on less productive soils (Appendix A).

### Data

#### Discharge and Water Quality

Measurements of river discharge (daily values) and water quality (grab samples: on average 33 per year) are collected in national monitoring programs (Niemi 2006). The data are available at Syke Open data portal (https://www.syke.fi/opendata).

The data are produced and collected mainly by national and regional environmental administrators (The Finnish Environment Institute Syke and Centers for Economic Development, Transport, and the Environment). The sampling times vary in every catchment but, on average, there is one sample per month with some exceptions in spring months, when there may be 2–4 samples for each of those months.

#### Meteorological and Deposition Data

Meteorological data and the atmospheric deposition data were obtained from the open data of the Finnish Meteorological Institute (FMI). Deposition was available only from the year 1990 onwards to 2022.

#### Land Type Data

Digital Elevation Model (DEM) is produced by National Land Survey of Finland. Spatial data of forests with high conservation value was based on optimization done with Zonation programme (Mikkonen et al. 2023b). Both data sets are available at open database provided by Finnish Environment Institute (https://www.syke.fi/en-US/Open_information).

In this study we used national version 6 of the Zonation programme, in which the optimization is based on modelled dead wood potential, spatiotemporal information on forest management and drainage, observations of threatened forest species, and connectivity within forests and to protected areas (Mikkonen et al. 2020; Mikkonen et al. 2023b).

Soil data originated from the Finnish Soil Database (Lilja et al. 2006). Land cover was obtained from the CORINE Land Cover dataset (years 2000, 2006, 2012 and 2018). It has been produced by the Finnish Environment Institute (Syke), based on satellite images and data integration with existing digital map data, provided by the Natural Resources Institute Finland (Luke). The dataset provides information on Finnish land use and land cover, and its changes, including forest cut and forest gain area. There is also more detailed information of forest structure available in the Multi-Source National Forest Inventory (MS-NFI) data. (https://www.luke.fi/fi/seurannat/valtakunnan-metsien-inventointi-vmi).

We aggregated the Corine land cover classes into the following categories: Agri (agricultural land), ForMin (economical forests on mineral soil), ForOrg (economical forests on organic soil), ForMinCut (forest cuts on mineral soil), ForOrgCut (forest cuts on organic soil), OldFor (forest older than 80 years).

### Simply-C Model and its Set-up

The family of the Simply models includes parsimonious models, incorporating a rainfall-runoff model and a biogeochemical model able to simulate daily streamflow and nutrient/carbon dynamics (Jackson-Blake et al. 2017). The Simply-C model tries to find the impact that SO4 deposition and climate variables have on the TOC concentration in river water. There is an option to calibrate several landcover classes separately to see their effect on TOC concentration. The model is dynamic, working on a daily time step, and is spatially semi-distributed i.e., there is the ability to differentiate between hydrology and carbon processes, and between forest use types and sub-catchments (with associated stream reaches). The models are available at the Mobius platform (Norling et al. 2021). To summarize, in each land use class and each sub-catchment the soil water DOC concentration is modelled to reach an equilibrium of

$${[\text{DOC}]}_{s}={\left[\text{DOC}\right]}_{0}\left(1+\,{k}_{T}{T}_{s}-{k}_{{{SO}}_{4}}{[\text{SO}}_{4}]\right)$$

where ${\left[\text{DOC}\right]}_{0}$ is a land-use specific equilibrium concentration of DOC in soil water when soil temperature is 0 °C, and ${k}_{T}$ and ${k}_{{{SO}}_{4}}$ are linear coefficients of the response of DOC to soil temperature ${T}_{{s}}$ and ${\text{SO}}_{4}$ deposition respectively. The runoff from all the land use classes is mixed as they reach the river (potentially with different delays) and can be diluted if precipitation is heavy.

We used data of the closest meteorological and deposition measurement station for each river basin (Fig. 1). The Simply-C model was calibrated against measured river discharge and measured TOC concentration in the river water. The calibration period was 1985-–2019. The parameter values were first tuned manually, and then by automatic optimization. TOC fluxes from different land use and land cover classes were then checked to correspond with literature values (Sallantaus 1992; Piirainen 2002; Finér et al. 2003; Mattsson et al. 2005; Kortelainen et al. 2006; Liski et al. 2006; Palviainen et al. 2014; Lehtonen et al. 2016a; Manninen et al. 2018; Nieminen 2004) and measurements from small catchment studies (https://metsainfo.luke.fi/fi/vesistokuormitukset). Calibration criteria for discharge was N-S (Nash-Shutcliffe efficiency; Nash & Sutcliffe 1970) and KGE (Kling-Gupta efficiency; Gupta and Kling 2011), and for TOC Bias (Percent Bias; Gupta et al., 1999), and RMSE (Root Mean Square Error; Gupta et al. 1999) (Table 2). In addition, residuals were checked to be normally distributed and random (Appendix B and C). In Finnish rivers over 90% of TOC is in dissolved form (Kortelainen 1993). COD observations were transformed to TOC concentrations by formulas presented by Kortelainen (1993), but only in the Kemijoki River data, which had only few TOC observations.

**Table 2:** Goodness-of-fit values of observed and simulated discharge (Q) and DOC concentrations

Code	Name	Mean Q [m^3^ s^−1^]		Goodness-of-fit Q		Mean DOC [mg l^−1^]		Goodness-of-fit DOC	
		Observed	Simulated	KGE	N-S	Observed	Simulated	Bias	RMSE
11	Virojoki	4.41	3.93	0.744	0.626	16.29	15.33	−0.133	9.257
16	Koskenkylänjoki	8.62	6.72	0.64	0.177	10.59	15.36	−3.129	8.249
18	Porvoonjoki	12.67	9.86	0.734	0.534	11.528	13.505	−1.519	7.333
19	Mustijoki	6.734	6.404	0.713	0.458	14.693	14.693	−0.379	7.29
21	Vantaanjoki	16.307	15.469	0.79	0.61	12.19	14.076	−1.616	7.769
24	Kiskonjoki	5.90	5.688	0.782	0.562	10.423	11.926	−1.46	4.902
27	Paimionjoki	9.432	8.569	0.762	0.584	13.094	13.62	−0.318	7.131
28	Aurajoki	7.555	7.129	0.882	0.565	14.487	15.297	−0.578	8.206
34	Eurajoki	9.377	8.624	0.502	0.132	10.632	11.794	−1.417	6.496
37	Lapväärtinjoki	14.268	12.13	0.637	0.41	16.936	16.531	2.59	9.913
44	Lapuanjoki	33.48	31.142	0.768	0.665	20.537	19.935	1.907	10.886
47	Perhonjoki	23.934	21.941	0.784	0.614	20.702	20.819	1.838	12.263
51	Lestijoki	12.789	9.035	0.716	0.403	20.674	18.853	3.098	10.002
53	Kalajoki	38.442	26.416	0.537	0.381	17.22	21.594	5.682	9.064
54	Pyhäjoki	33.923	14.546	0.678	0.534	17.692	16.845	1.598	8.033
57	Siikajoki	42.952	43.275	0.706	0.392	20.038	16.738	3.403	8.011
60	Kiiminginjoki	45.391	51.456	0.721	0.595	15.1	12.789	2.043	8.155
61	Iijoki	174.716	176.018	0.634	0.424	10.813	12.311	−1.989	8.59
64	Simojoki	42.36	41.707	0.74	0.625	11.36	13.194	−1.658	8.041
65	Kemijoki	572.739	632.714	0.69	0.269	7.785	9.413	−1.363	5.227

According to the KGE the calibration of the discharge was acceptable in all rivers and based on N-E value at least satisfactory in most of the rivers (Moriasi et al. 2007; Moriasi et al. 2015). The general level of simulated and observed TOC concentrations were at the same level (RMSE). Bias was low, indicating very good calibration.

### Scenarios

#### Scenario 1: Climate Change

We used daily data from five global climate models (CMIP5; Climate model intercomparison project) under representative concentration pathway (RCP) scenarios RCP4.5 and RCP8.5, which we statistically downscaled onto a high-resolution grid (approximately 10 km x 10 km) using a quantile-mapping method (Lehtonen et al. 2016b). The models were chosen based on their ability to simulate present-day average monthly temperature and precipitation climatology in northern Europe and the availability of all required variables (air temperature, precipitation, net shortwave radiation, relative humidity, cloud cover) on a daily timescale. Simulations over the historical period until 2005 were combined with simulations under RCP4.5 and RCP8.5 emission scenarios for the period 2006–2099. The RCP8.5 (Riahi et al. 2011) is a high-emission scenario leading to a warming of global land areas by almost 5 ˚C by 2100. In the RCP4.5 scenario (Thomson et al. 2011) the warming on the global scale is about half of that in the RCP8.5 scenario. The average temperature and precipitation change over Finland are presented in Appendix D.

**Fig. 2:**
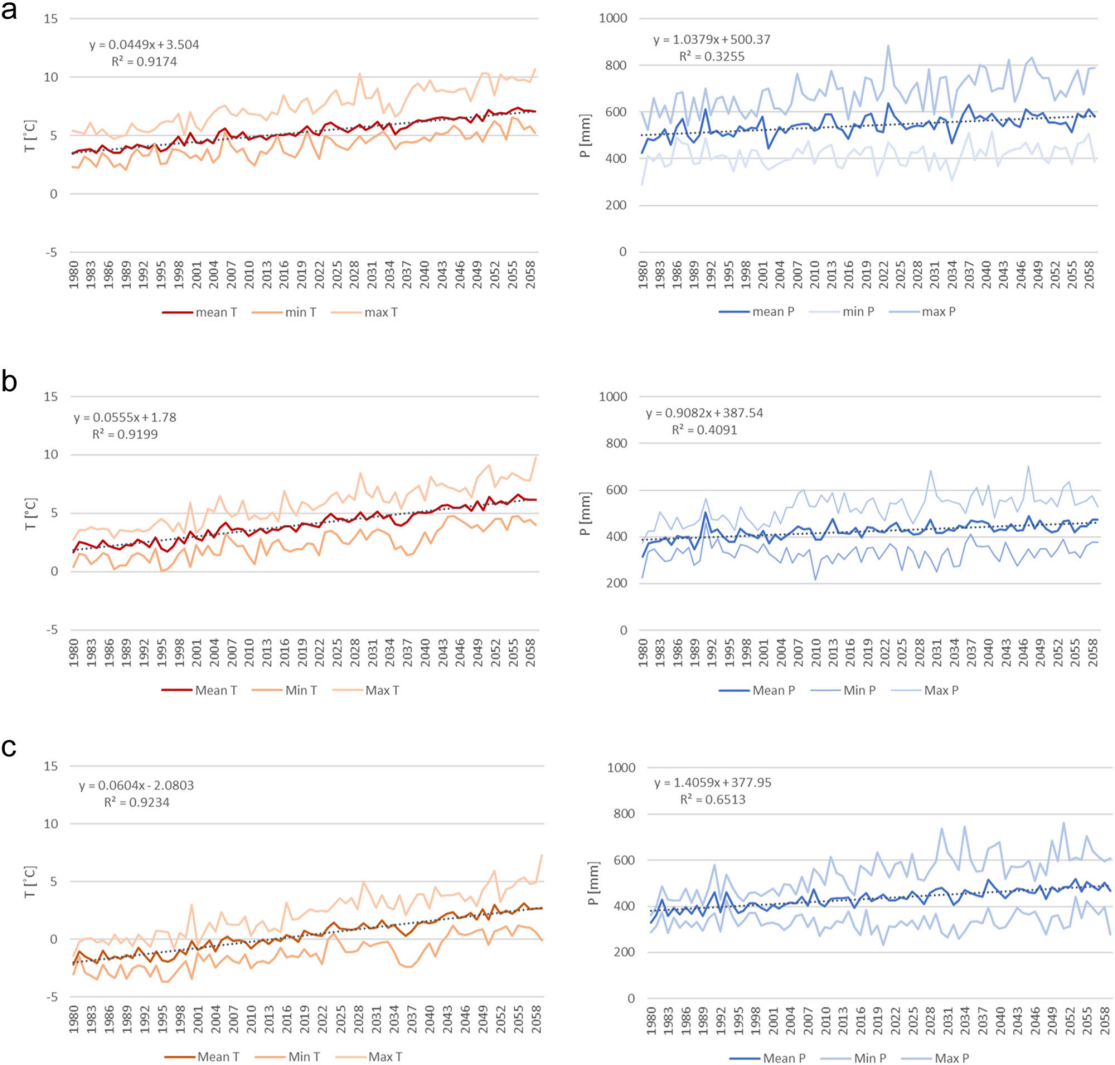
Increase in temperature and precipitation (mean, minimum and maximum of five global climate models; 1980–2060) at different meteorological stations (**a**) Jokioinen Observatory, (**b**) Kauhava Airport, (**c**) Rovaniemi Airport. Locations of the stations are shown in Fig. 1

The annual average air temperature predicted by the models increased statistically significantly. In the north, the temperature rose faster than in the south (Fig. 2). Precipitation increased the most in the north. The dispersion between the models increased towards the end of the period (Appendix D).

#### Scenario 2: Atmospheric Deposition å

For atmospheric SO_4_ deposition we used the chemical transport model results that are based on the EMEP MSC-W model (v4.4) and the MATCH model results (Engardt et al. 2017). The data is available at the Zenodo repository (metno/emep-ctm: OpenSource rv4.36 (202011)). The atmospheric deposition scenarios were undertaken to support the EU 7th Framework project ECLAIRE - Effects of climate change in air pollution impacts and response strategies for European ecosystems (http://www.eclaire.ceh.ac.uk/).

**Fig. 3:**
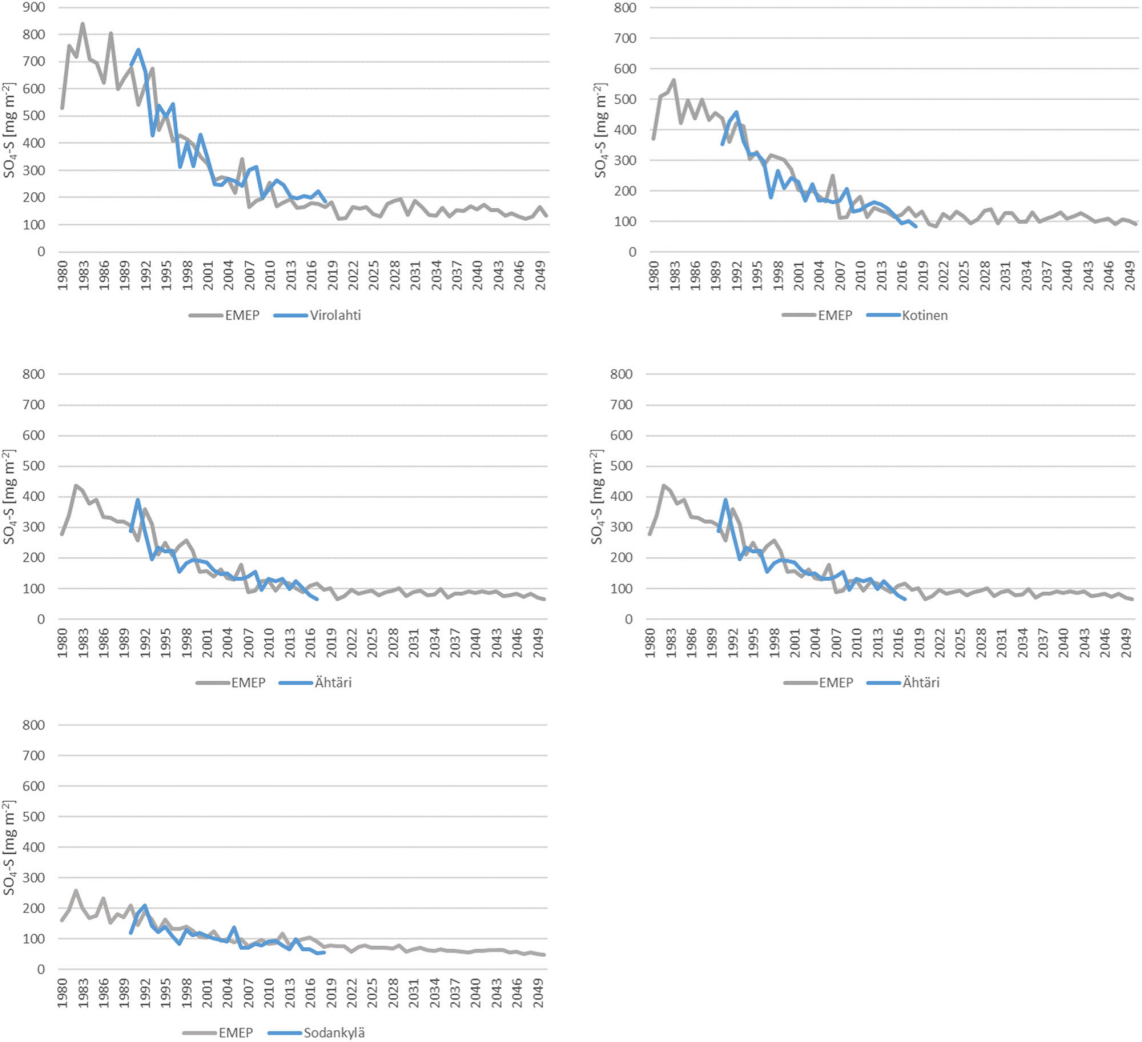
Observed sulphur deposition at five observation sites (location shown in Fig. 1) and simulated (EMEP/MATCH) sulphur deposition at corresponding EMEP grid. Locations of the stations are shown in Fig. 1

The gridded deposition data (50 km × 50 km) corresponded well to the level and trend of measured deposition at the Finnish observation sites (Fig. 3). The historical deposition was highest in south-east Finland and lowest in Lapland. There was a clear decrease at all the stations which levelled off around 2015, and the low level is predicted to continue in future scenarios.

#### Scenario 3: Forest use Change

We explored two forest use scenarios: (1) forest management, and (2) forest protection. Both changes took place on forest land. In the analysis we did not include changes in land use between forest and other land use forms.

**Fig. 4:**
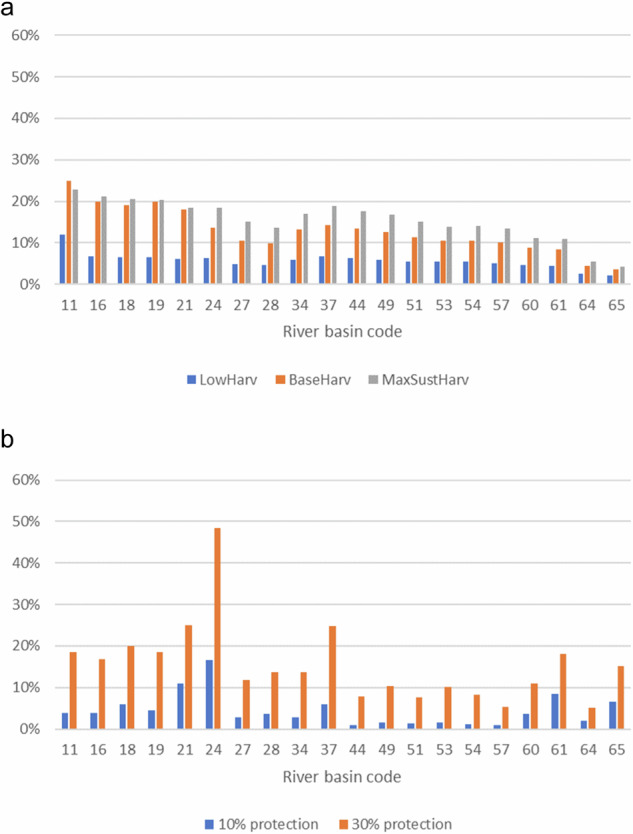
Mean forest use change in different scenarios in Finland (**a**) influential forest cutting area, (**b**) forest protection area

The forest management scenario was based on simulations with the PREBAS forest growth and carbon balance model (Mäkelä et al. 2023), where forests were thinned or clear-cut following Finnish national recommendations (Äijälä et al. 2019) but restricted regionally not to exceed a long-term maximum sustainable harvest so that productivity and profitability will be maintained in the long term (the intensity of harvest is not greater than the forest growth). Forest felling was assumed to have around 10 years influence time on water quality (Finér et al. 2010). Thus, the area of influence (Fig. 4) was clearly larger than the area of annual forest fellings. Current realized harvests were close to this (Base scenario). In Low scenario forest harvest area was half of the area of maximum sustainable cuts.

The following transitions between the forest use classes were allowed. No changes in area of agriculture (Agri), and changes between areas of forest on mineral soil (ForMin) and fellings on mineral soil (ForMinCut); changes between areas of forest on organic soil (ForOrg) and fellings on organic soil (ForOrgCut); changes between ForMin/ForOrg and old forest (OldFor).

In the forest protection scenario, 10 and 30% of the forests with highest conservation values in Finland were set aside, respectively, and no further harvesting was allowed in these areas. The most valuable forest areas for biodiversity were based on a spatial optimization model using the software Zonation (Mikkonen et al. 2018; Mikkonen et al. 2020; Mikkonen et al. 2023b, a). To identify the most valuable forests, we primarily employed data on forest structure and quality (vegetation class, tree species, volume and diameter), which provide ecologically useful surrogates for conservation value in boreal forest. The protected area was distributed across the river basins, southern Finland having the highest potential to increase protected forest areas (Fig. 4b).

We ran global scenarios of atmospheric deposition and different climate scenarios for the period 1990 to 2060 and took the mean of results (DOC export) as the baseline for DOC export. Then, we ran local scenarios for forest use change for the same period and compared these results (DOC export) to the baseline.

## Results

### Effect of Climate Change and Atmospheric Deposition on Brownification

Discharge, TOC concentration and TOC load showed a significant (Mann-Kendall statistics) increasing trend from 1980 to 2059 (Table 4). In TOC loading, the increase was on average 20–40%, but in the northernmost river basin it doubled.

**Fig. 5:**
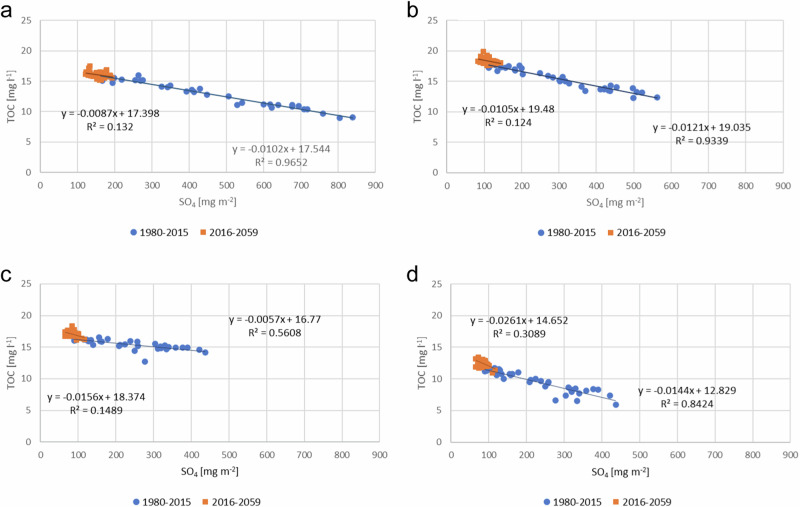
Correlation between S deposition and TOC concentration (**a**) Group 1, (**b**) Group 2, (**c**) Group 3, (**d**) Group 4. River basin groups are shown in Fig. 1 and Table 1

There was a correlation between decreasing deposition and increasing TOC concentration in all river basin groups (Table 3). Annual TOC concentrations were highest in peat dominated river basins, and the variation between the individual basins in this group was high. In peat-dominating river basins the increase seemed to be linear, in southern mineral soil dominated river basins it leveled off around 2015 (Fig. 5). Increase per year was higher in the period 1980–2015 than in 2016–2059 (Value of Q in Table 4). The increase per year was higher in period 1980–2015 than in 2016–2059 (Value of Q in Table 4). There was a strong correlation between SO_4_ deposition and TOC concentration in period 1980–2015, but that correlation weakened or disappeared in the period 2016–2059 (Fig. 5).

**Table 3:** The Pearson correlation coefficient between annual concentration change and anthropogenic pressures in different river basin groups

	Group 1	Group 2	Group 3	Group 4
Deposition	−0.980	−0.966	−0.866	−0.941
Air temperature	0.619	0.807	0.675	0.761

River basin groups are presented in Fig. 1 and Table 1

**Table 4:** Mann-Kendall statistics (Q = change per year, Z positive (negative)) value of Z indicates an upward (downward) trend

Time series	Unit	Area	Period	n	Z	Signific.	Q
Load	kg/day	Group 1	1980–2015	36	5.79	***	33.102
		Group 2	1980–2015	36	5.27	***	23.582
		Group 3	1980–2015	36	3.12	**	20.073
		Group 4	1980–2015	36	6.47	***	215.74
		Group 1	2016–2059	44	2.62	**	9.441
		Group 2	2016–2059	44	1.51		3.621
		Group 3	2016–2059	44	2.50	*	10.233
		Group 4	2016–2059	44	5.96	***	128.76
Runoff	mm	Group 1	1980–2015	36	2.71	**	1.149
		Group 2	1980–2015	36	2.33	*	0.724
		Group 3	1980–2015	36	1.62		0.591
		Group 4	1980–2015	36	2.63	**	0.874
		Group 1	2016–2059	44	2.56	*	0.944
		Group 2	2016–2059	44	0.98		0.278
		Group 3	2016–2059	44	1.20		0.207
		Group 4	2016–2059	44	1.87	+	0.448
Concentration	mg/l	Group 1	1980–2015	36	6.55	***	0.196
		Group 2	1980–2015	36	6.61	***	0.154
		Group 3	1980–2015	36	5.90	***	0.056
		Group 4	1980–2015	36	6.69	***	0.144
		Group 1	2016–2059	44	1.37		0.011
		Group 2	2016–2059	44	5.59	***	0.040
		Group 3	2016–2059	44	6.73	***	0.039
		Group 4	2016–2059	44	8.14	***	0.042

*** 0.001 level of significance

** 0.01 level of significance

* 0.05 level of significance

+ if 0.1 level of significance

**Fig. 6:**
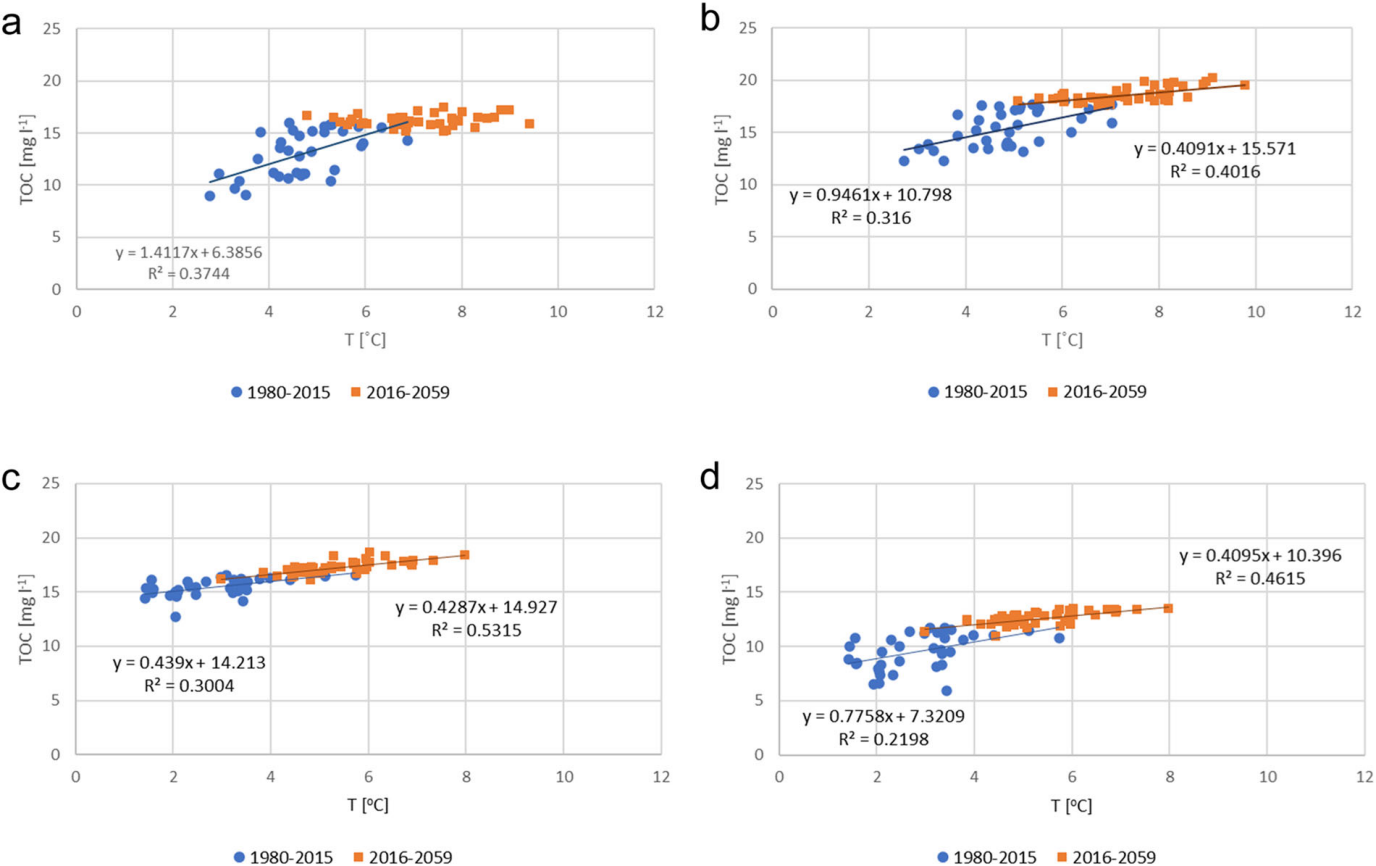
Correlation between air T and TOC concentration (**a**) Group 1, (**b**) Group 2, (**c**) Group 3, (**d**) Group 4. River basin groups are shown in Fig. 1 and Table 1

There were correlations between air T and TOC concentration in all groups (Table 3). Correlation between TOC concentration and air T became statistically more significant in peat-dominated (Group 3) and northern (Group 4) river basins in 2016–2059 but disappeared in southern catchments (Fig. 6).

**Fig. 7:**
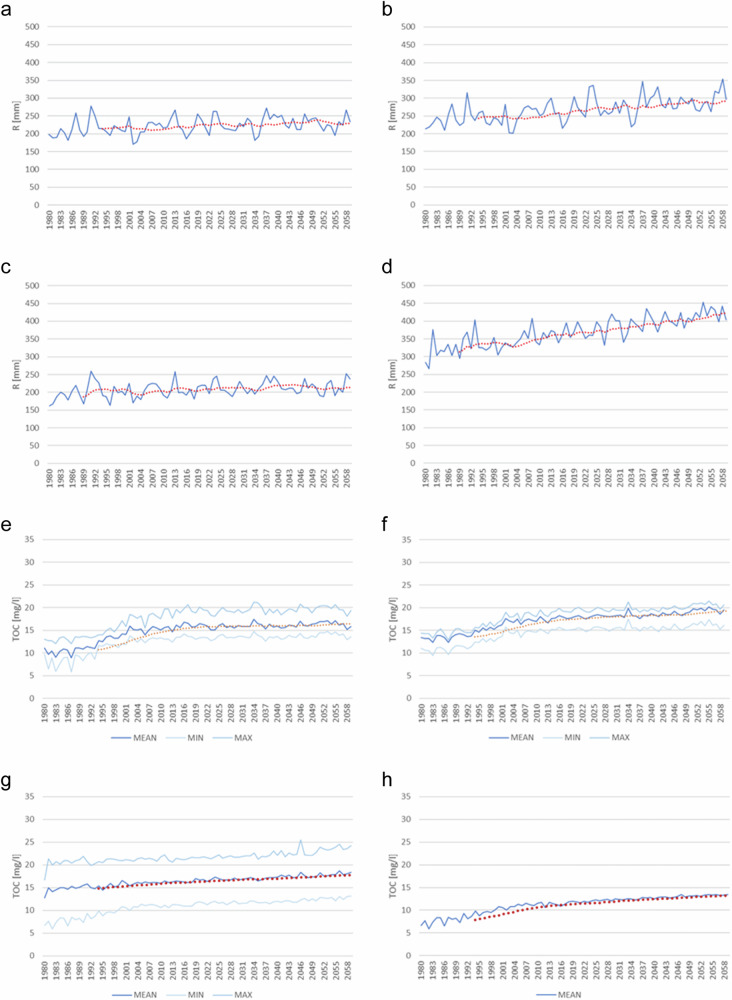
Mean, minimum and maximum runoff in different river basin groups (**a**) Group 1, (**b**) Group 2, (**c**) Group 3, (**d**) Group 4, and mean TOC concentration (**e**) Group 1, (**f**) Group 2, (**g**) Group 3, (**h**) Group 4. River basin groups are shown in Fig. 1 and Table 1

There was a variation in annual runoff within all river basin groups (Fig. 7a–d). Runoff was highest in the northernmost river basin. When the whole period of 1980–2059 was divided into two (1980–2015 and 2016–2059), the significance of trend disappeared in ‘Group 3’ river basins (Table 4). In other groups both significance and value of Q (increase per year) were higher in the period 1980––2015 than in 2016–2059.

Loading of TOC from all river basin groups showed a statistically significant increasing trend (Table 4; Fig. 7e–h) in the period 1980–2015. In the period 2016–2059 the increase was not significant any more in river basin ‘Group 2’. Again, increase in loading per year was lower in the period 2016–2059 than in 1980–2015. In the latter period TOC loading increased in ‘Group 1’ by 14%, in ‘Group 3’ by 6% and ‘Group 4’ by 28%.

### Effect of Forest use Change on Brownification

**Fig. 8:**
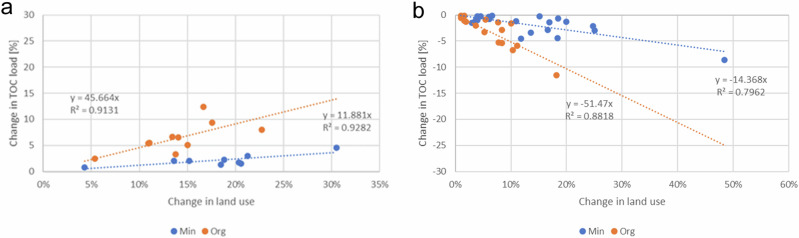
Simulated effect of forest use on TOC loading (**a**) scenario of forest cutting, (**b**) scenario of forest protection

Alternative forest management and protection schemes had a clear influence on TOC loading from river basins, and the effect was more pronounced in river basins that were dominated by organic soil types than in river basins dominated by mineral soils. The relationship between the percentage of forest use change (both forest cutting scenario and forest protection scenario) in the basin and the percentage of change in TOC load was statistically significant (Fig. 8).

There was a positive relationship between harvest level and run-off, but also between harvest level and concentration. The increase in the TOC load in mineral soil-dominated river basins remained low even in river basins with relatively large felling areas. In river basins dominated by organic soils the increase of the TOC loading was clearly higher.

In the forest protection scenario, we assumed that the chosen forests areas were protected, i.e., they were left untouched and developed towards the structure of a forest in the over 80 years age group with higher volume and basal area. Thus, runoff from old forests decreased due to higher evaporation. The reason for the decreased TOC load from river basin was mainly due to a change in runoff.

## Discussion

TOC concentration and loading from river basins increased in the climate and atmospheric deposition scenarios. The simulated increase in loading was leveling off around 2015 in southern river basins but not in the northern river basin.

The simulated increase in TOC concentration correlated well with both decrease in atmospheric deposition and with an increase in temperature in the period 1980–2015. According to the EMEP scenarios, atmospheric deposition decreased during this period, and then levelled off. This is also observed by Cano Bernal et al. (2022) in statistical studies of long time series. The strong correlation between TOC concentration and atmospheric deposition weakened in the period 2016–2059 in all river basin groups. Atmospheric deposition did not change much post 2016, so then it is no longer a driver of TOC.

According to most climate change scenarios, the temperature will increase until 2070, but the precipitation will only increase approximately by 10% of the current level (Appendix D). During 1980–2015, air T and TOC concentration correlated in all river basin groups. This correlation disappeared in southern river basin groups in 2016–2059 but continued in northern rivers. The observed mean temperature has already risen over 2 °C during the years 1847–2013, amounting to a 0.14 °C rise per decade (Mikkonen et al. 2015). This is consistent with human-induced global warming. At the same time, there were only low increase in annual precipitation. This is in line with the observation of Hohenthal et al. (2014) that precipitation has decreased rather than increased. According to the scenarios, both temperature and precipitation increased in the north more than in the south. Higher precipitation does not necessarily lead to greater runoff, as higher temperatures increase evaporation. In addition, the characteristics of the catchment area affect how much runoff is generated in the area (Mustonen 1986).

In simulations forest use change had a clear influence on brownification, especially in peat dominated river basins. Forestry measures, especially forest ditching on soils, increase brownification (Finér et al. 2021; Nieminen et al. 2021). In our study the simulated increase may be underestimated, as we assumed the effect of forest management to last only 10 years, but its effect may be even longer (Nieminen et al. 2018b). We also assumed the forest harvesting to be based on traditional clear cuts, not for example continuous cover forestry which may be more environmentally friendly solution (Nieminen et al. 2018a). DeLuca and Hatten (2024) concluded that land management practices with minimal soil disturbance and surface exposure are sufficient to achieve a meaningful conservation goal, while at the same time they meet human needs.

We did not divide the forests into deciduous and coniferous areas. Especially spruce-dominated forests are connected to higher TOC concentrations (Mattsson et al. 2005; Skerlep 2021). In a summary of the development of Finnish forests, spruce’s proportion of the productive forest area is decreasing at landscape level (Korhonen et al. 2021). On the other hand, the area of old forests, which are typically coniferous-dominated mixed forests, decreased brownification, as observed by Cano Bernal et al. (2022).

In southern Finland, the decreased impact of SO_4_ deposition (which has been the major driver of past brownification), along with precipitation and climate change, on TOC loading in the future will allow the effects of forest use to become more visible.Brownification is difficult to control by traditional water protection measures (Härkönen et al. 2023), so it is a positive signal that it can be influenced by land use planning. In addition to local policies, different EU policies require land use planning: EU Green Deal (European Commission, 2021a), EU Biodiversity Strategy 2030 (European Commission 2020) and Regulation on Land Use, Land Use Change and Forestry (LULUCF) (European Commission, 2021b).

Our results showed that extending forest protection to 30%could decrease brownification especially in areas where the influence of atmospheric pressure is decreasing. From a biodiversity point of view, the most valuable areas to protect are in the inland of Finland (Mikkonen et al. 2018; Appendix E), and the effect would firstly be seen in inland waters. The Finnish Nature Panel (an independent panel of scientific experts that supports nature and biodiversity policy planning and decision-making) has suggested that a 10% protection target should be implemented more equally in the country (Forsius et al. 2023).

Currently the area of forest cuttings is close to that of maximum sustainable forest cuttings. Mäkelä et al. (2023) have shown that increasing forest protection to match the EU 10% protection target would provide a significant carbon storage and sequestration potential by 2050, indicating complementarity of emission mitigation and conservation measures. In addition, it has favorable effects on surface water quality.

## Conclusions

Global atmospheric influence (atmospheric deposition and climate change) on brownification of surface waters seem to weaken in southern Finland after 2016. This allows more space for the effects of local changes in forest use due to EU policies.. Extending forest protection areas decreased brownification, especially in areas where the influence of atmospheric pressure is decreasing.When forest protection is planned to provide a carbon storage and sequestration potential and to favour biodiversity, it has favourable effects also on surface water quality. Land cover is more influential in river basins dominated by organic soils than in mineral soils. Browning still continues in northern Finland, mainly due to climate change.

## Supplementary information

Supplementary information

## Data Availability

No datasets were generated or analysed during the current study.
