# Supplementary material for: Modelling the Effects of Forest use Change on Brownification of Finnish Rivers under Atmospheric Pressure
Source: Environ Manage. 2024 Oct 18;75(2):205–20. doi: 10.1007/s00267-024-02058-1 (PMC11790703; doi:10.1007/s00267-024-02058-1)

APPENDIX A.

Basal area in different stand age a) Group 1, b) Group 2, 3) Group 3, 4) Group 4

a) b)


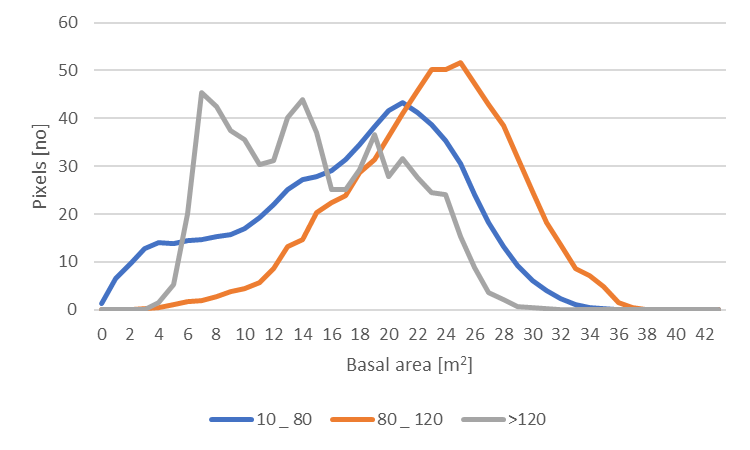

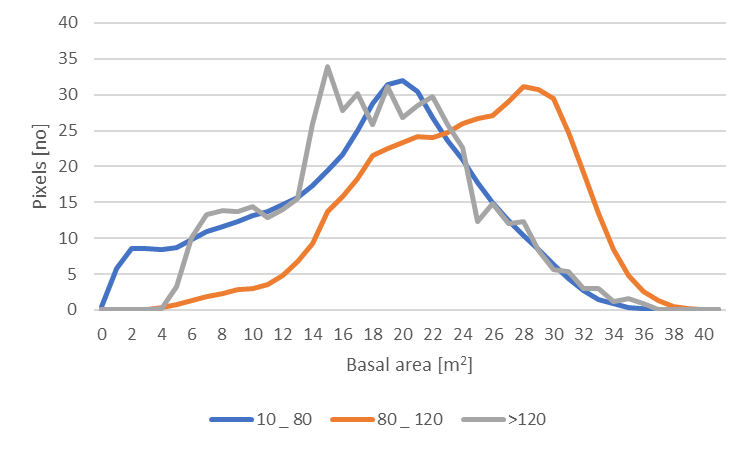


c) d)


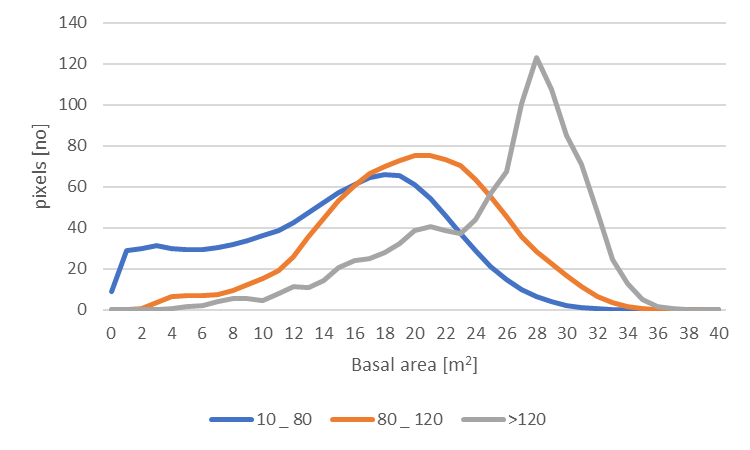

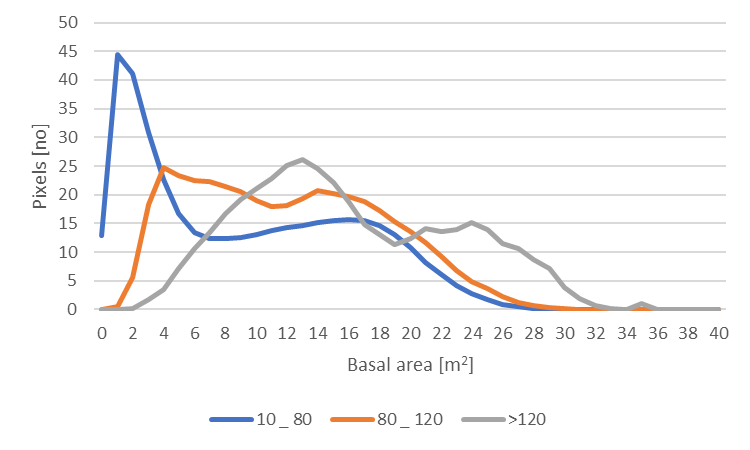


APPENDIX B.

Residuals of observed and simulated discharge in different rivers (number)

11


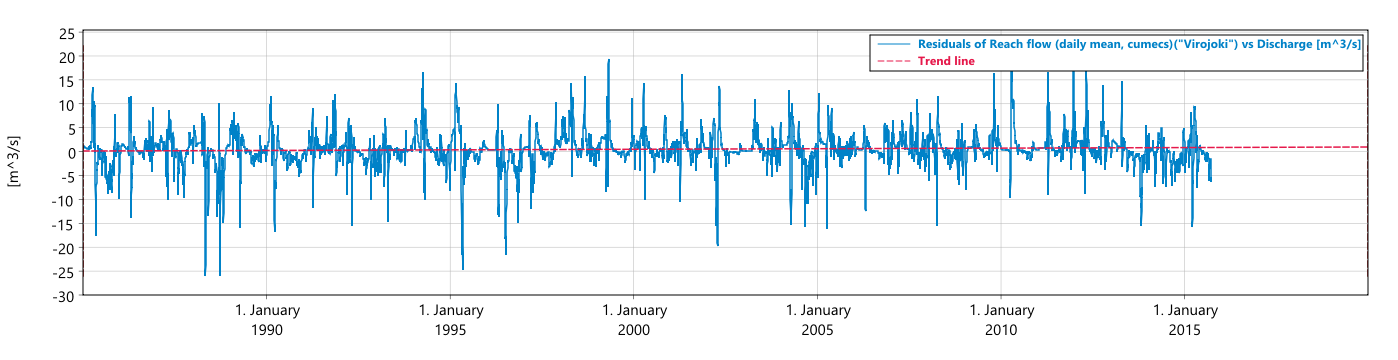


16


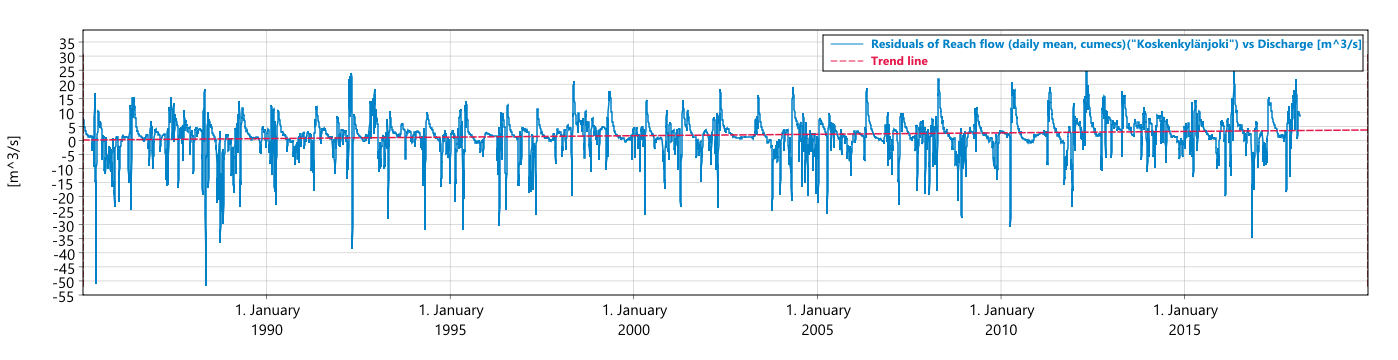


18


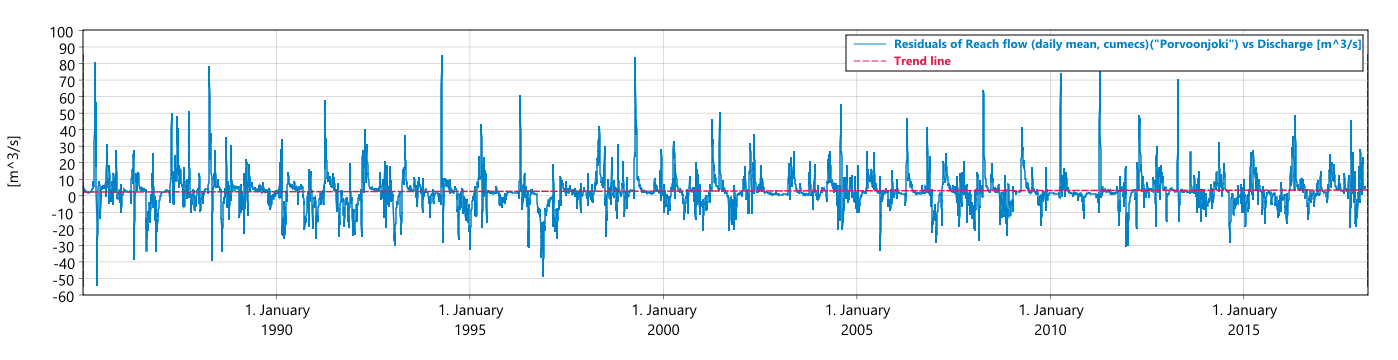


19


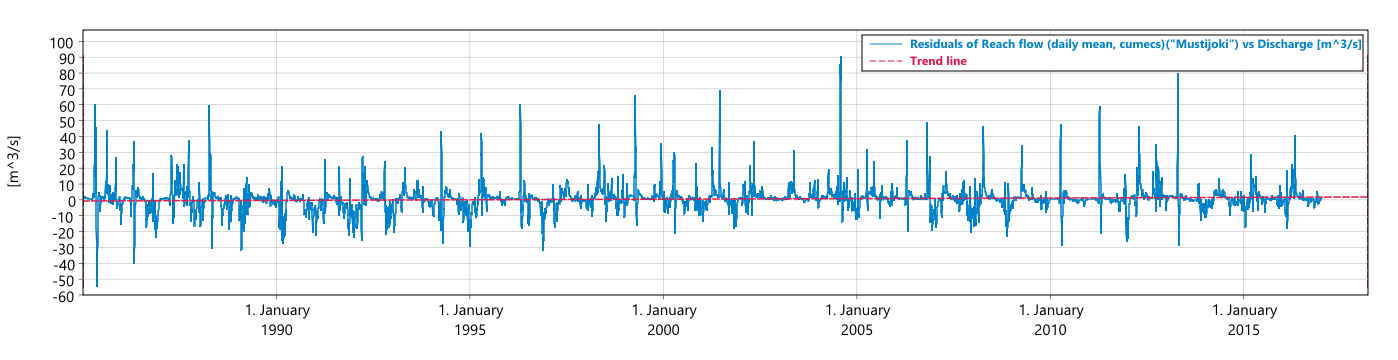


21


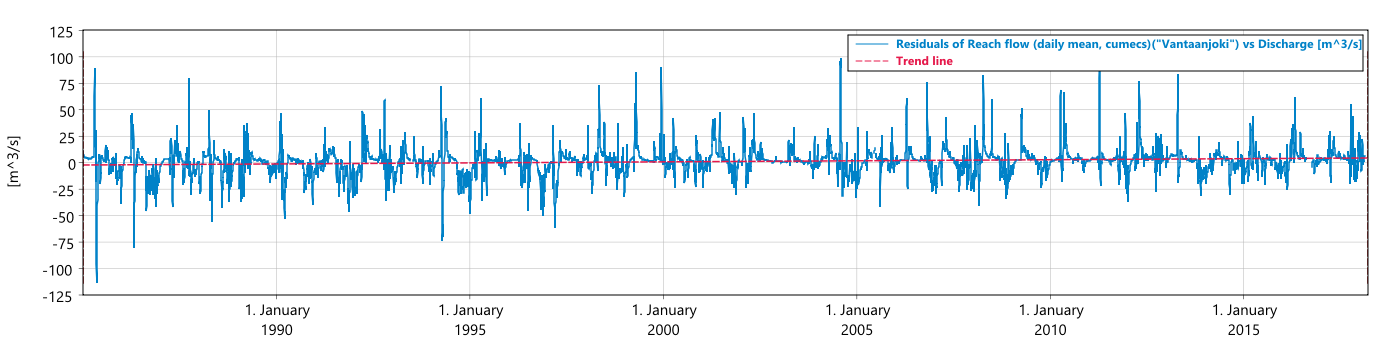


24


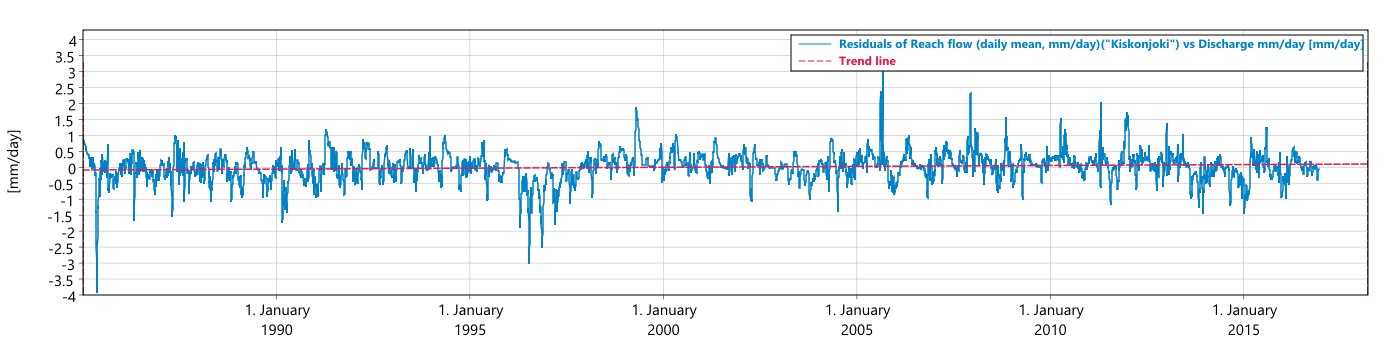


27


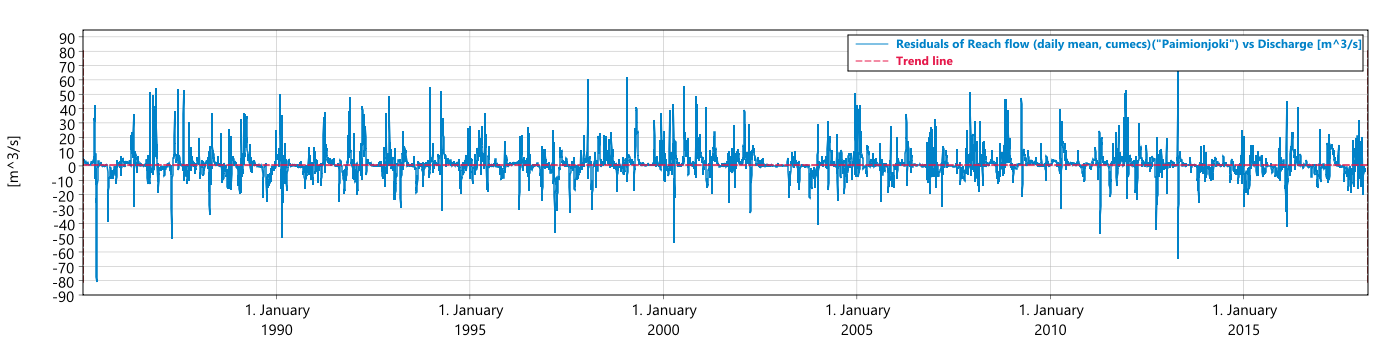


28


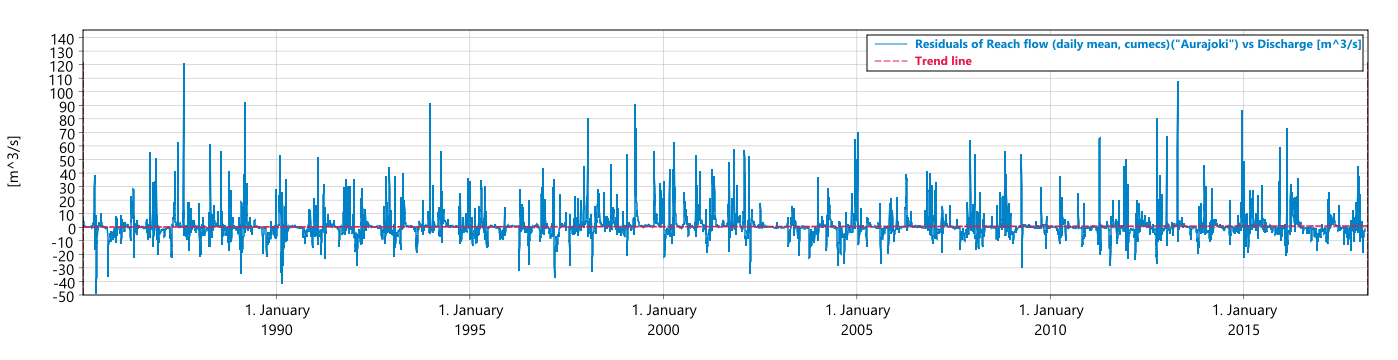


34


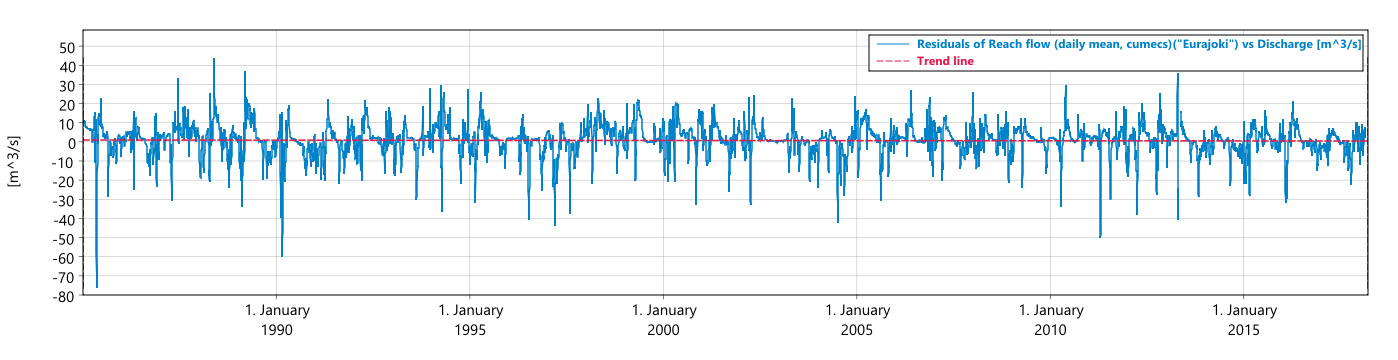


37


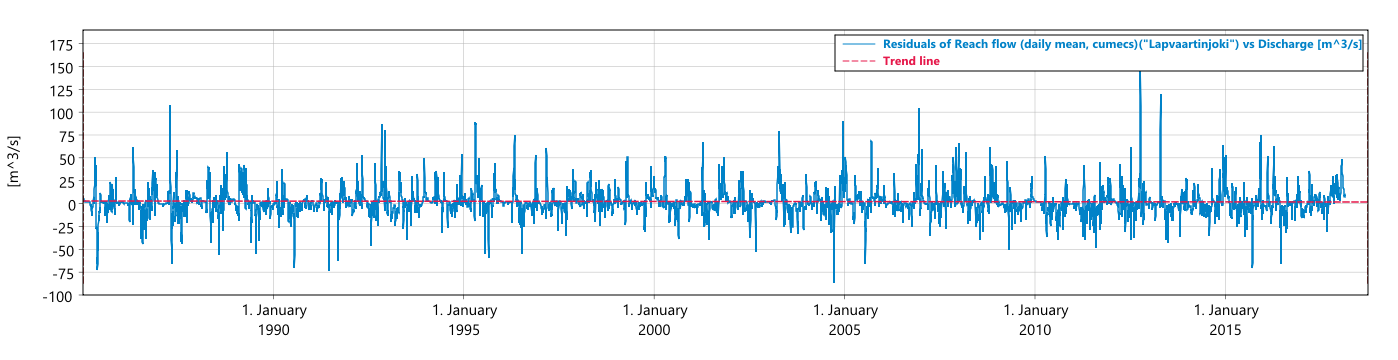


44


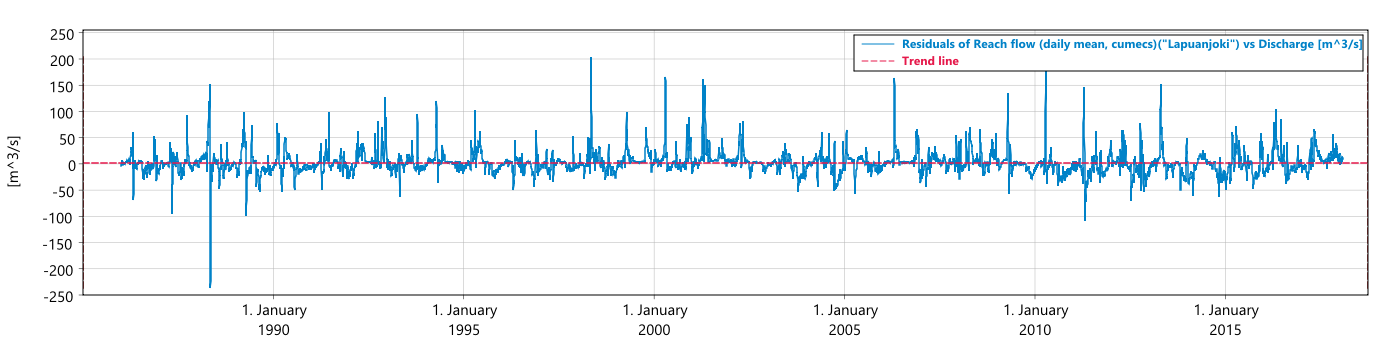


47


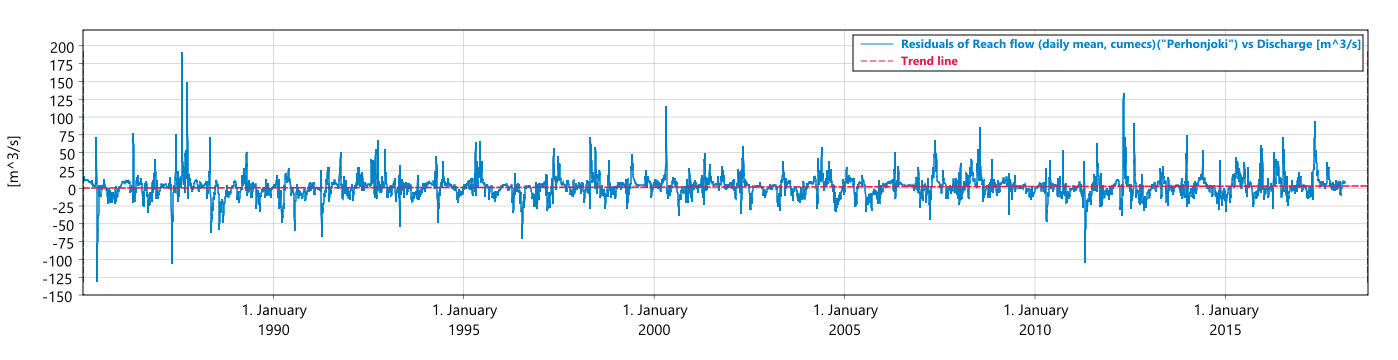


51


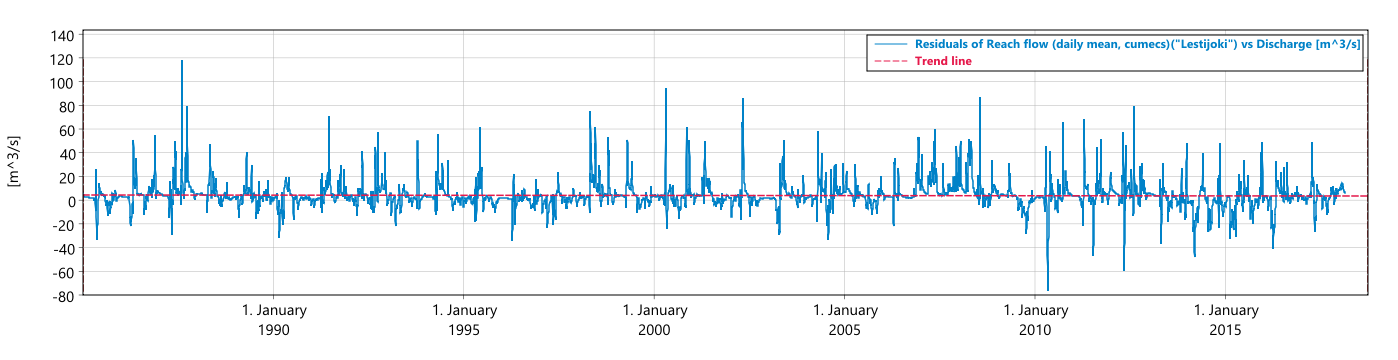


53


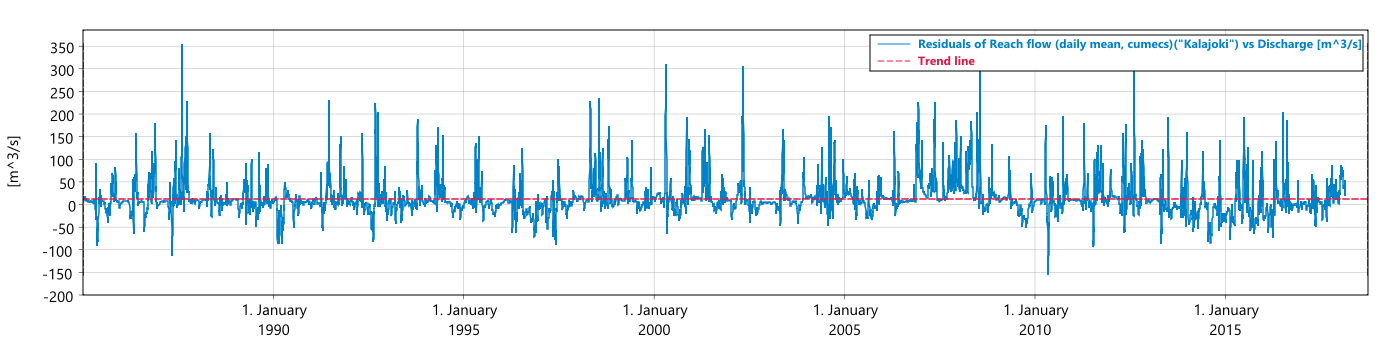


54


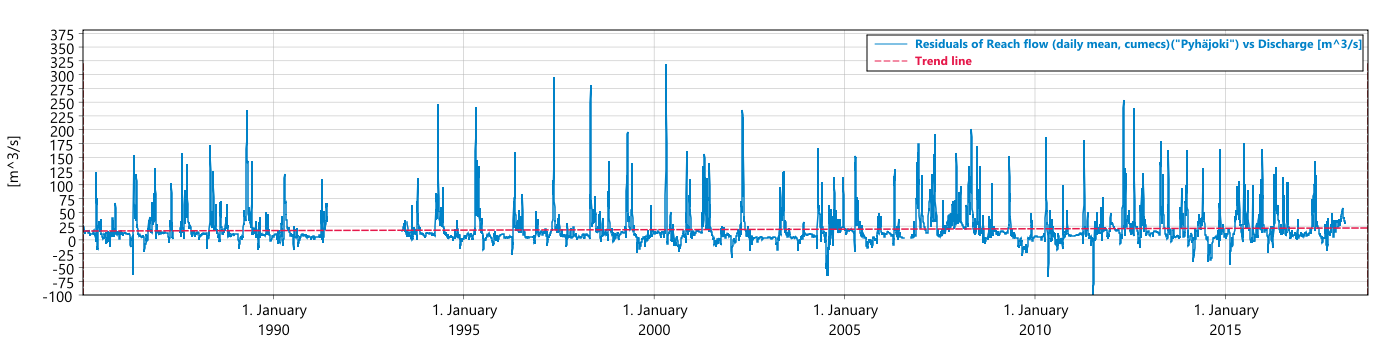


57


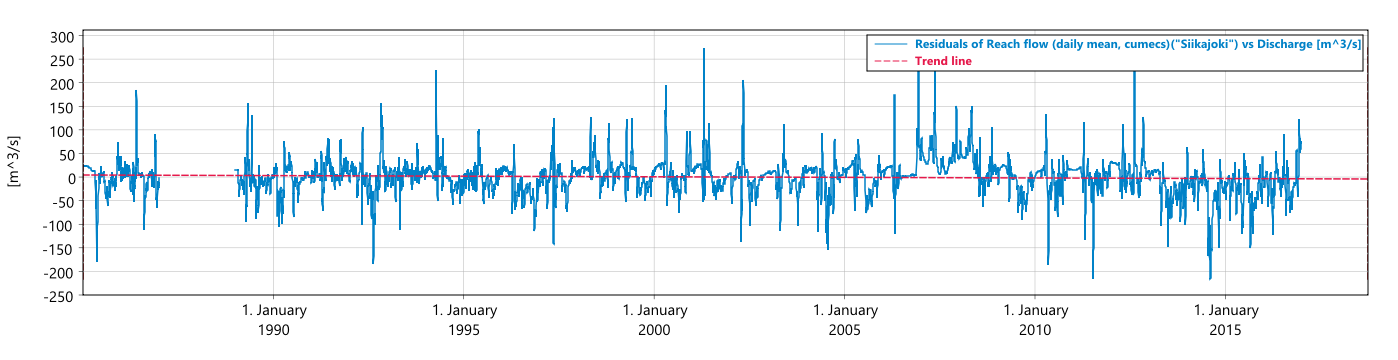


60


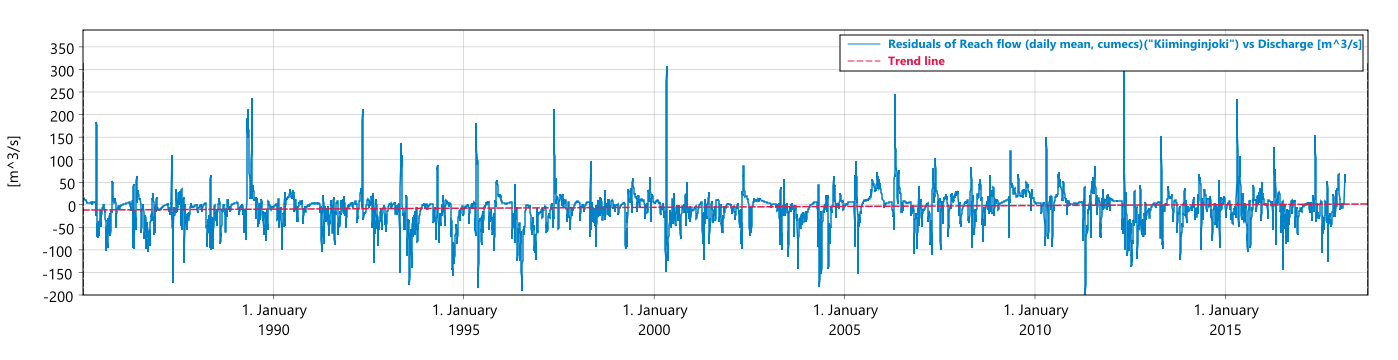


61


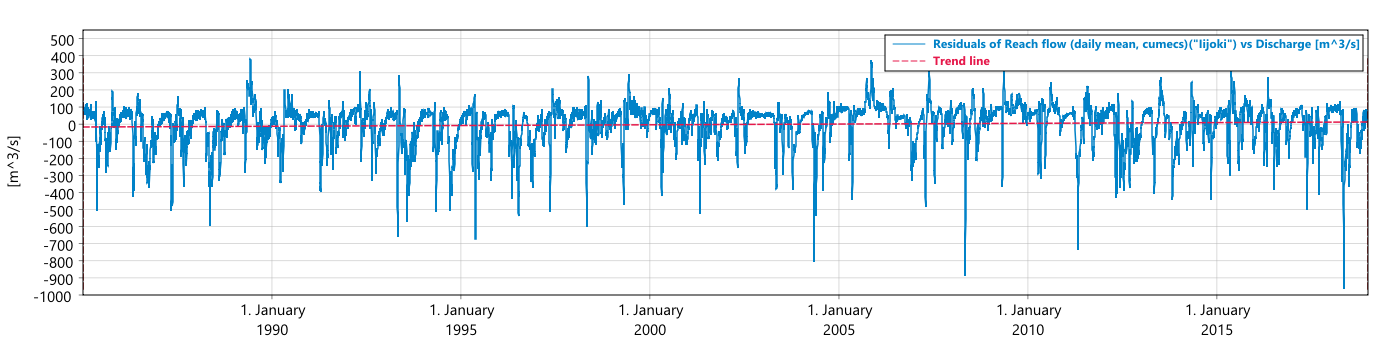


64


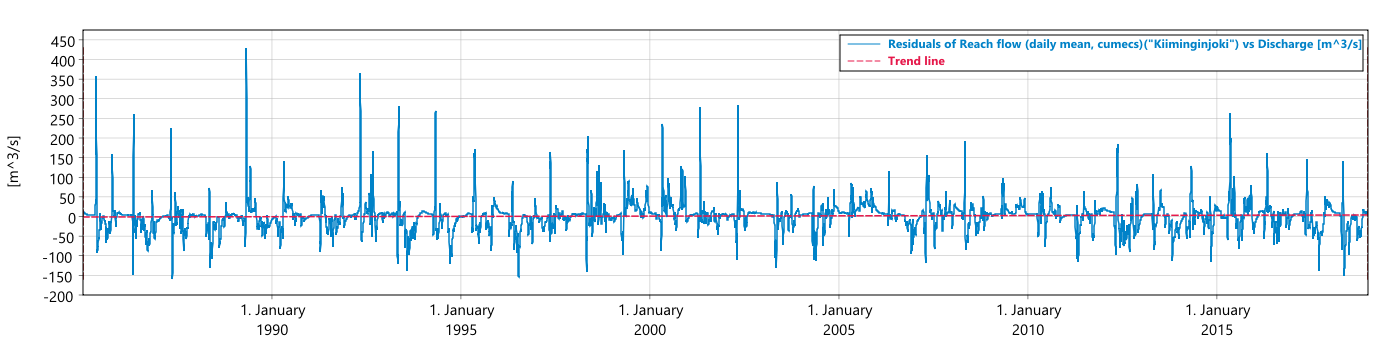


65


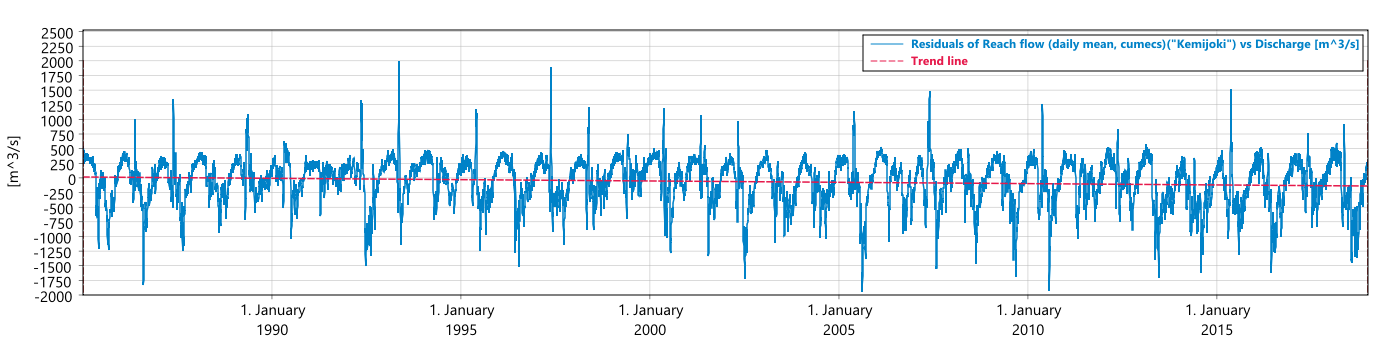


APPENDIX C.

Residuals of observed and simulated TOC concentration in different rivers (number)

11


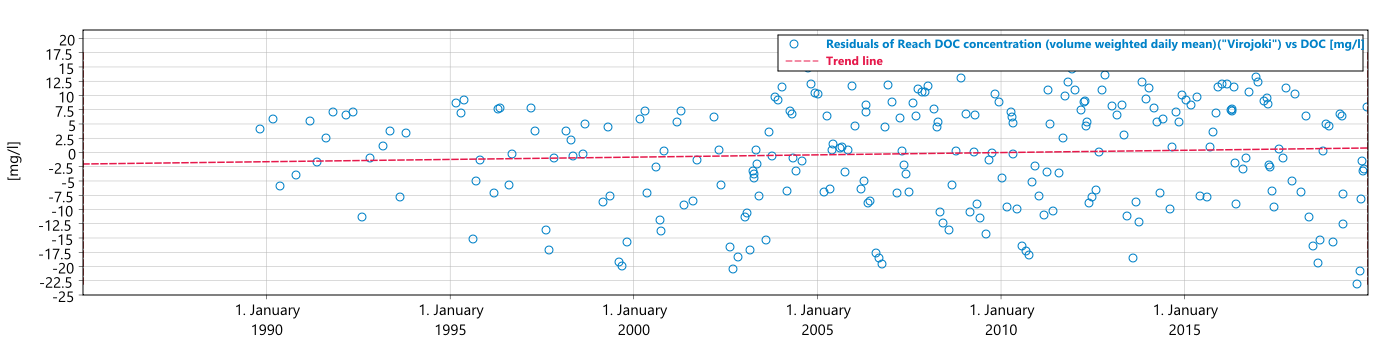


16


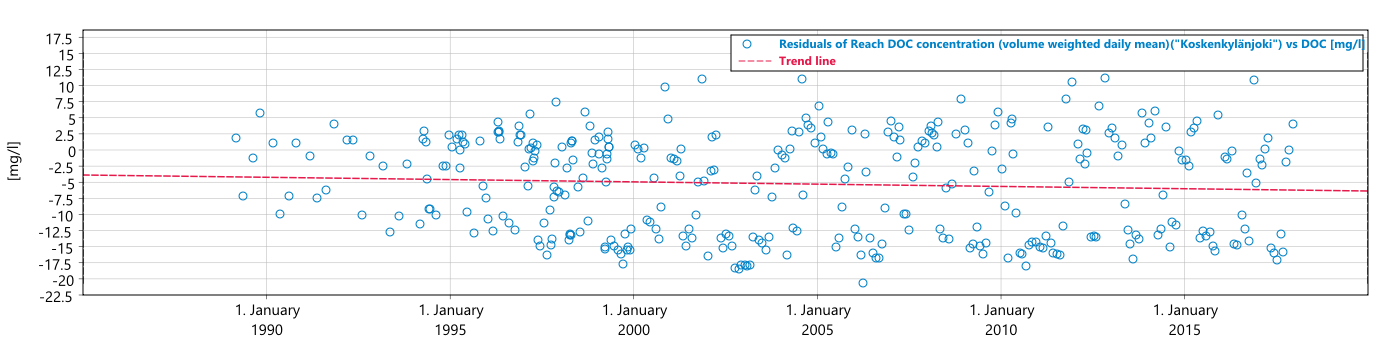


18


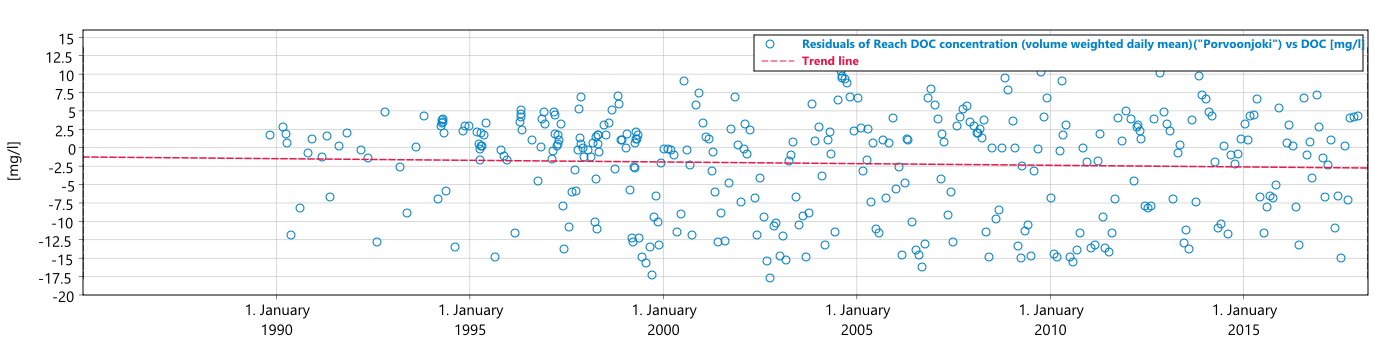


19


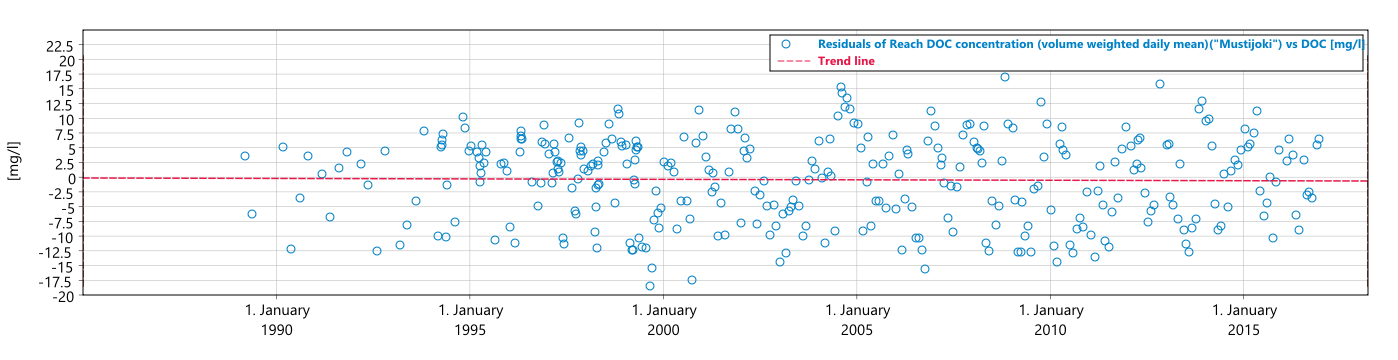


21


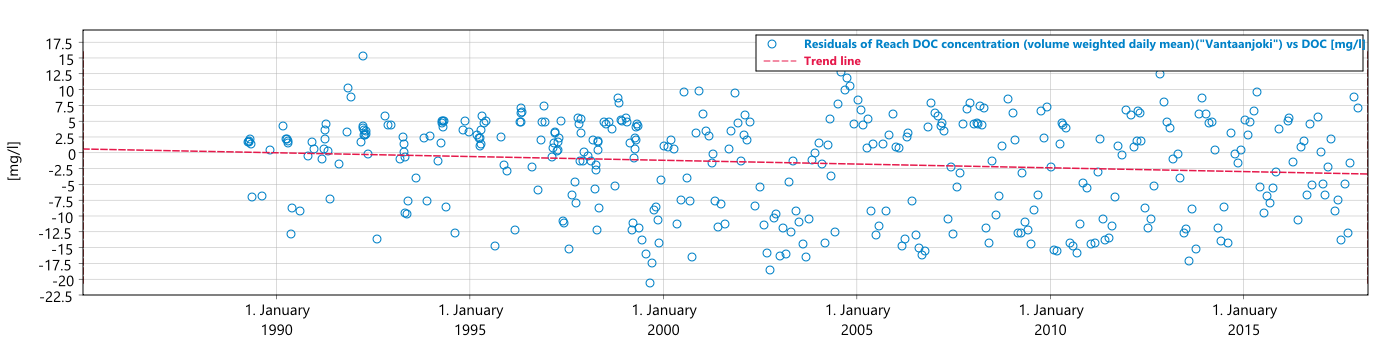


24


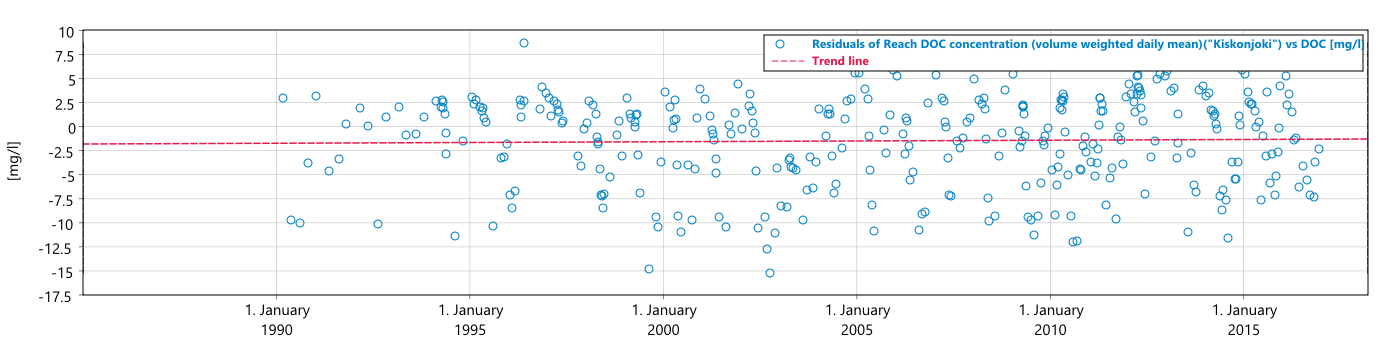


27


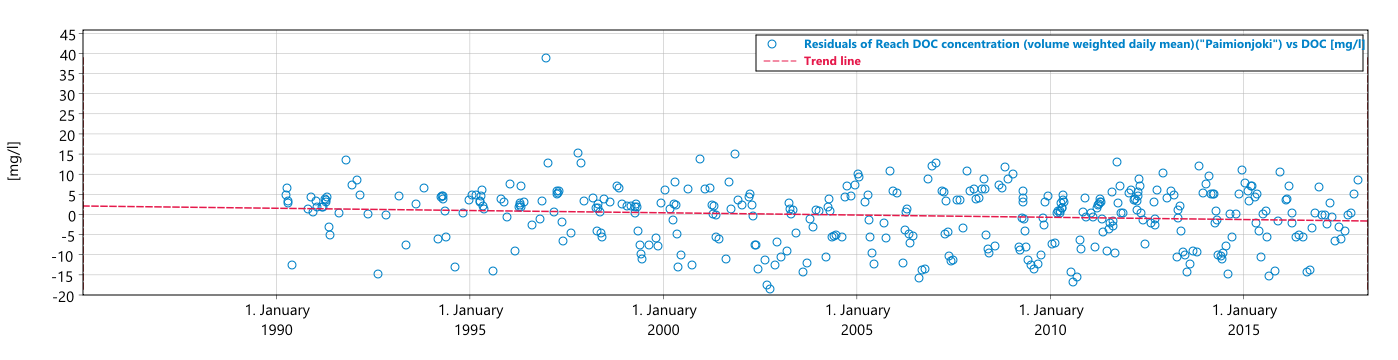


28


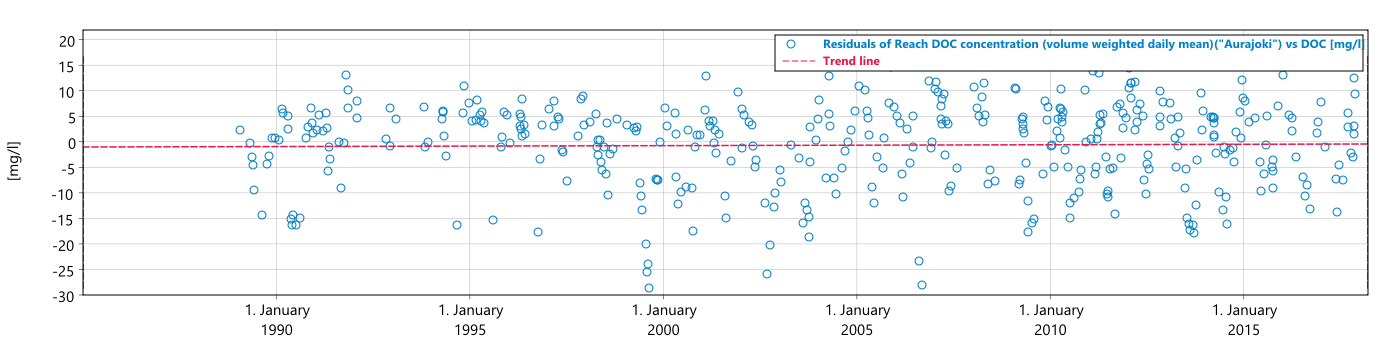


34


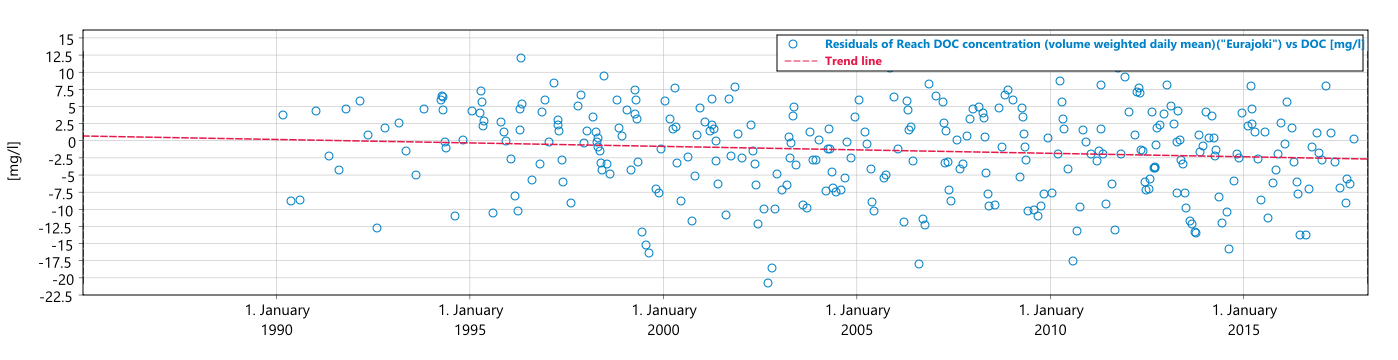


37


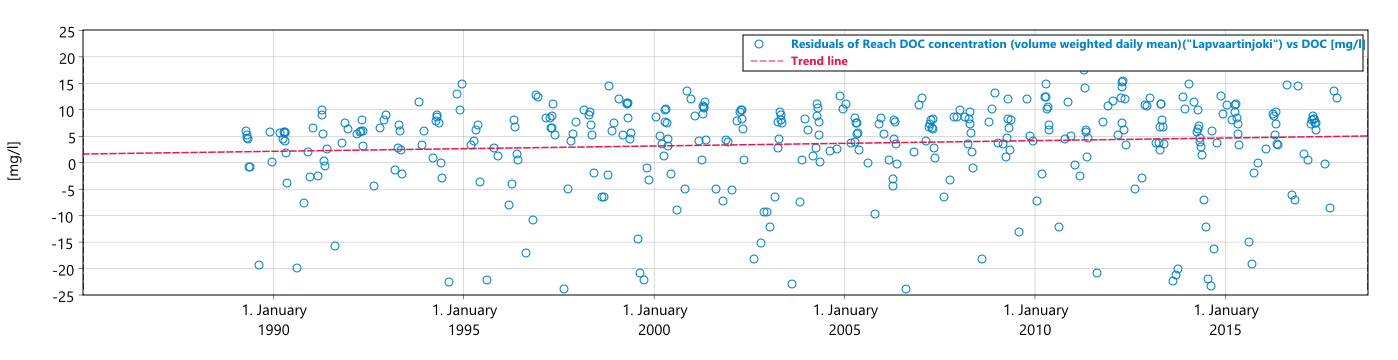


44


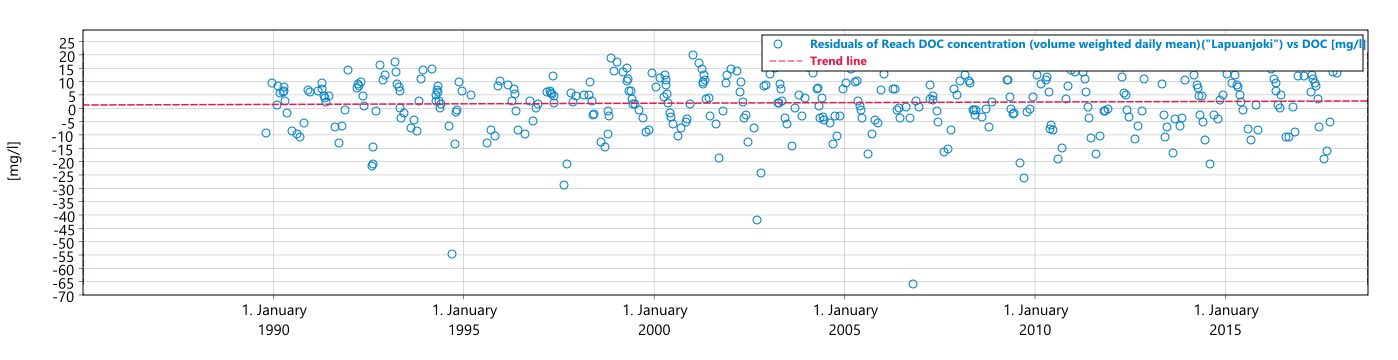


47


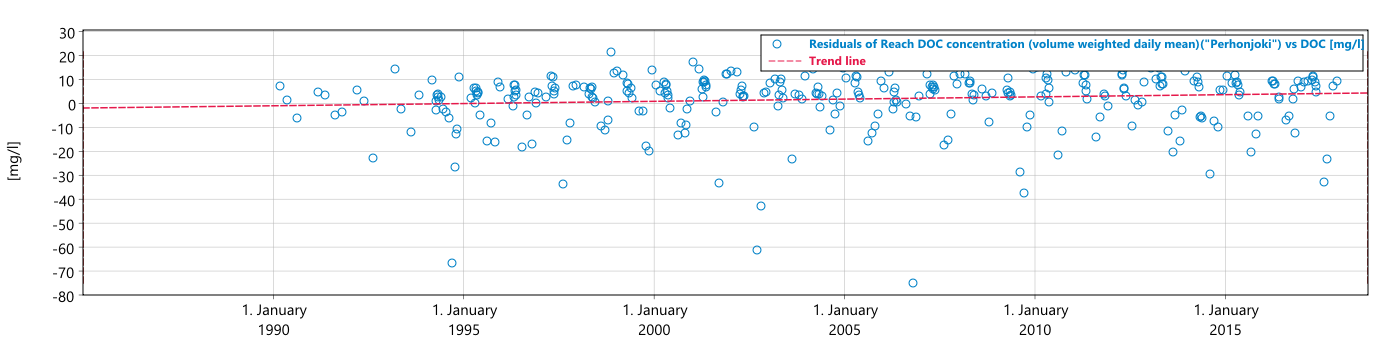


51


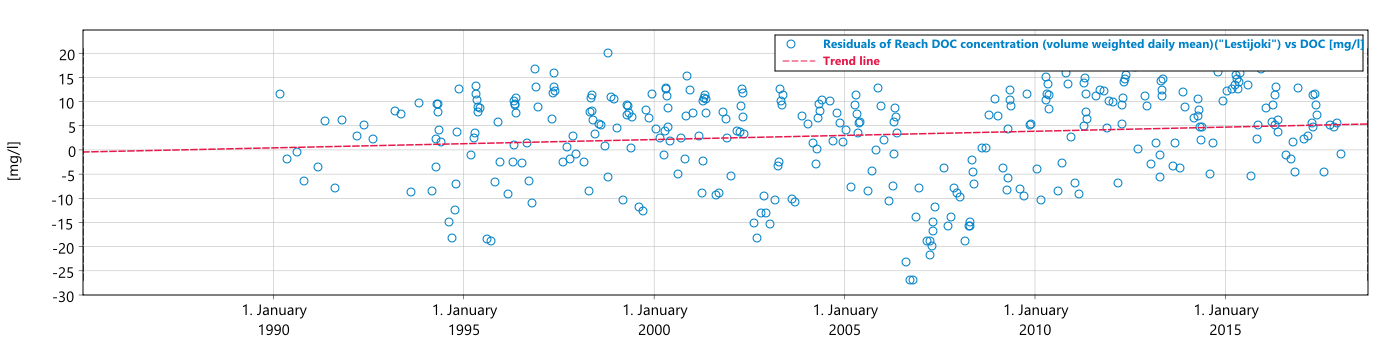


53


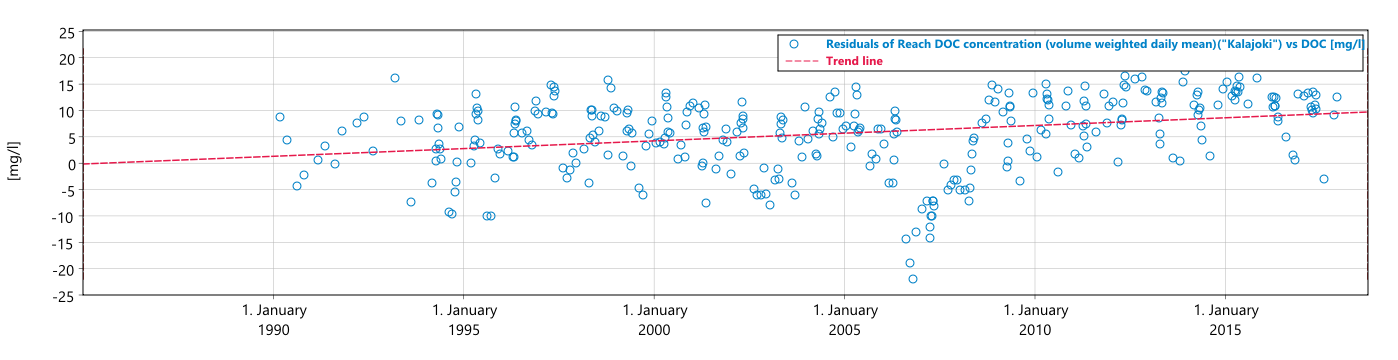


54


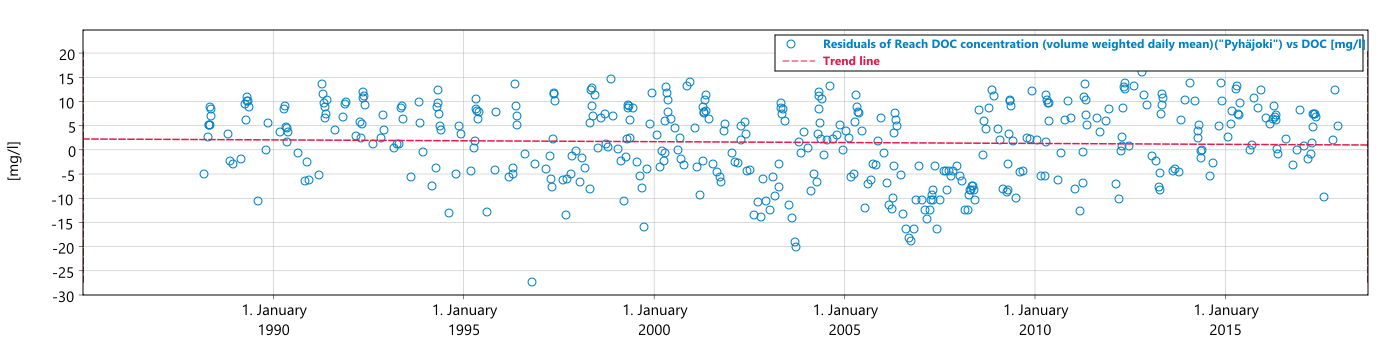


57


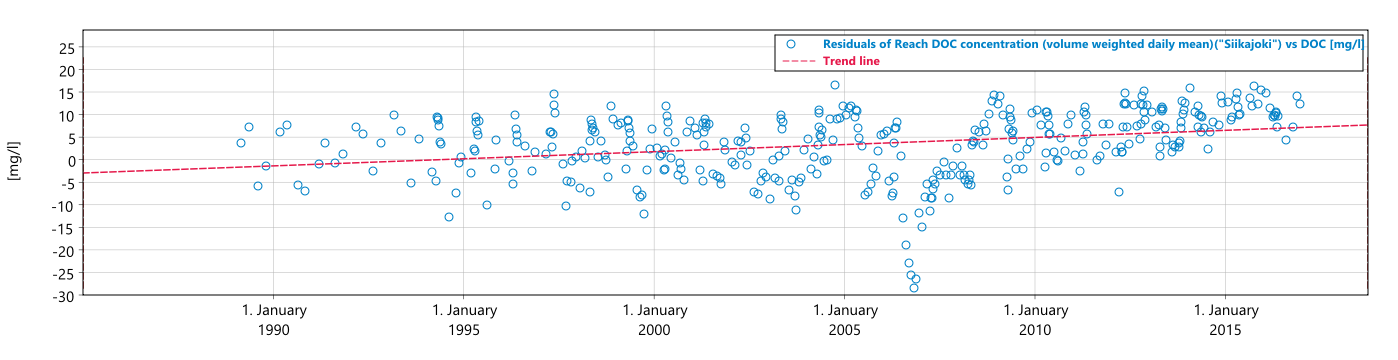


60


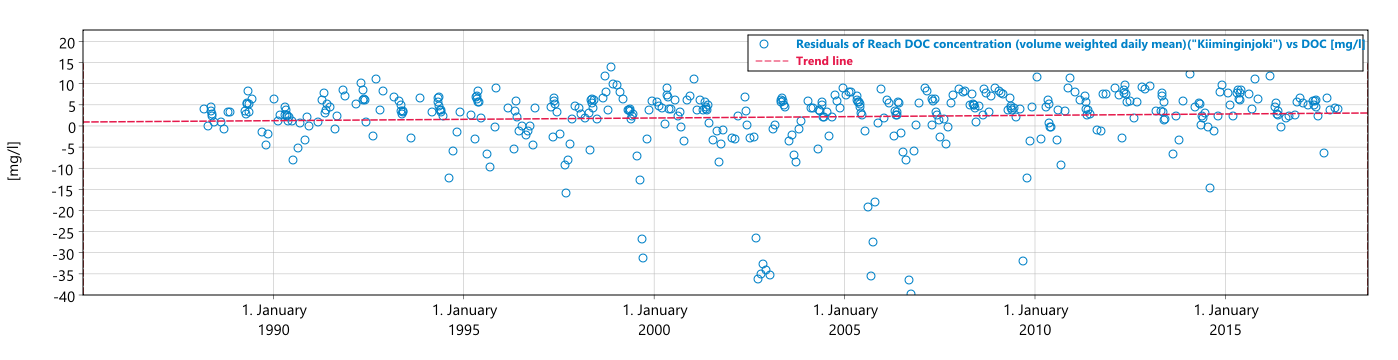


61


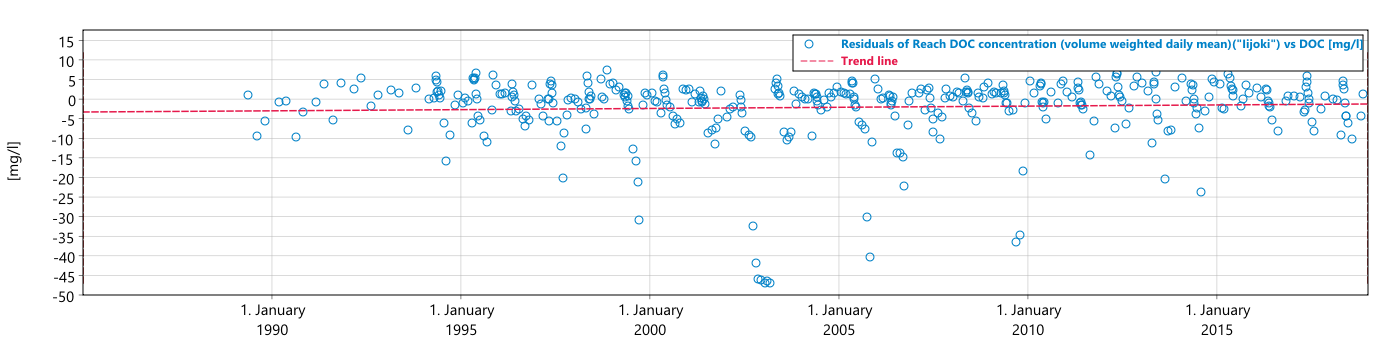


64


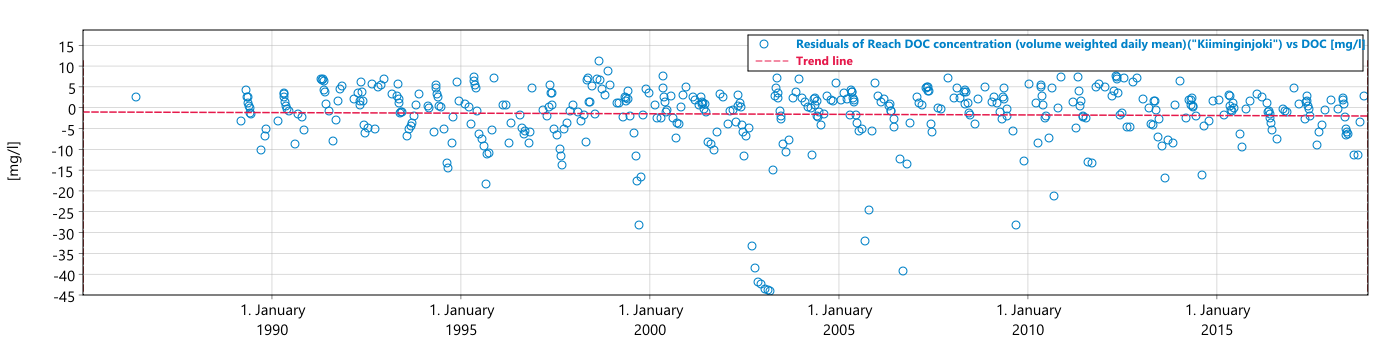


65


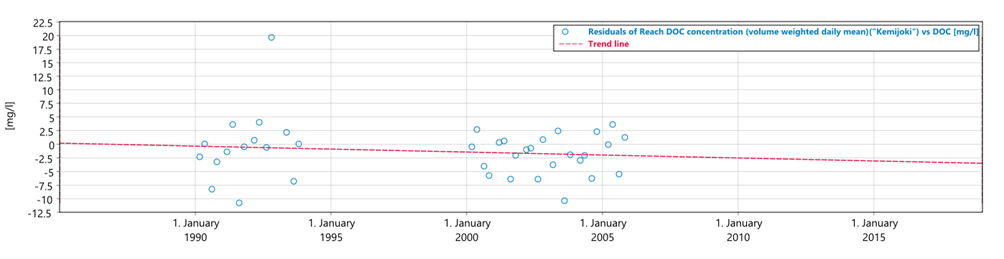


APPEDIX D.

Change in mean annual temperature (degrees of C) and precipitation (percentage) in Finland according to climate change scenarios


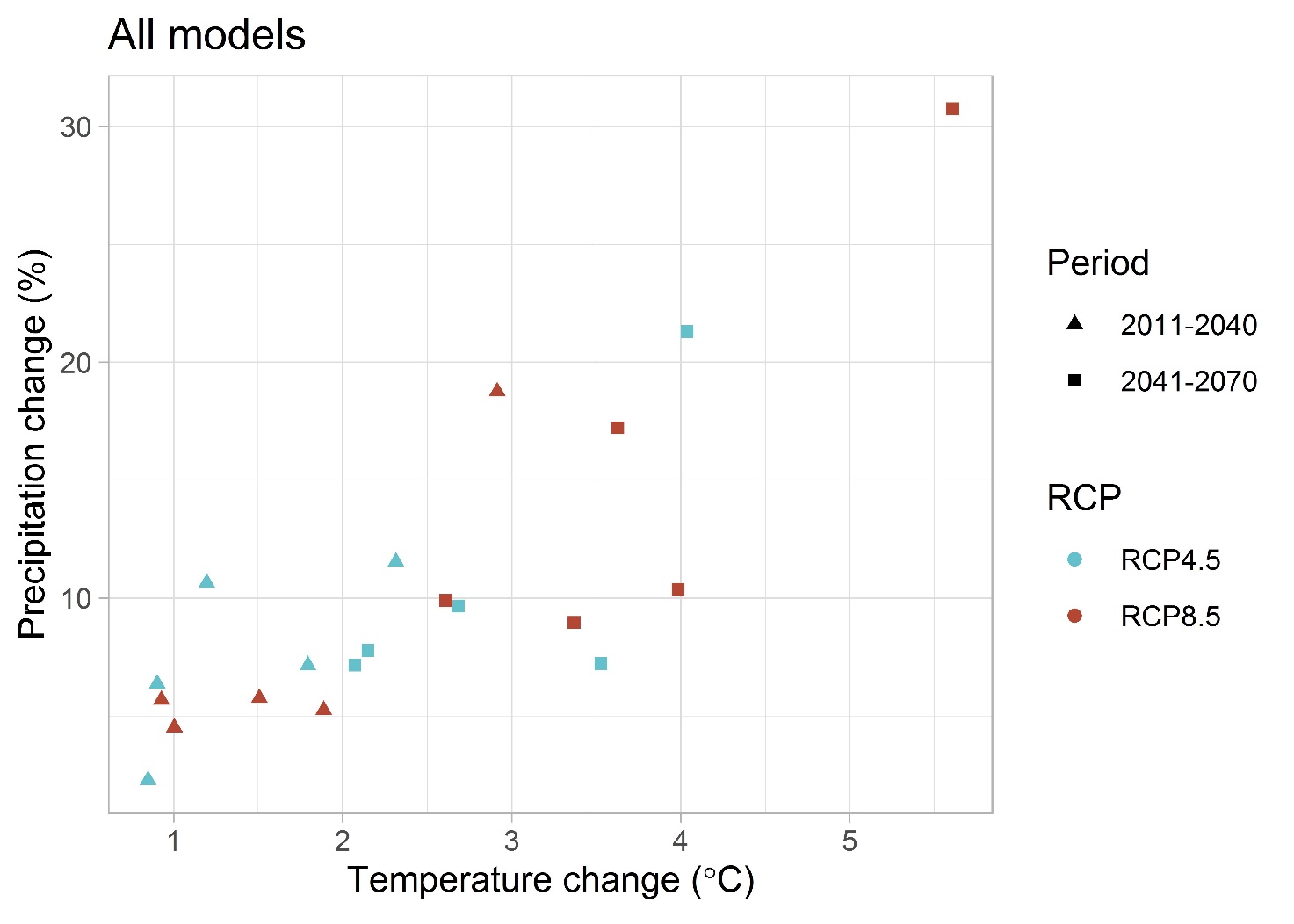


APPENDIX E.

Valuable forest areas for biodiversity according to Zonation analysis by Mikkonen N et al. (2018)


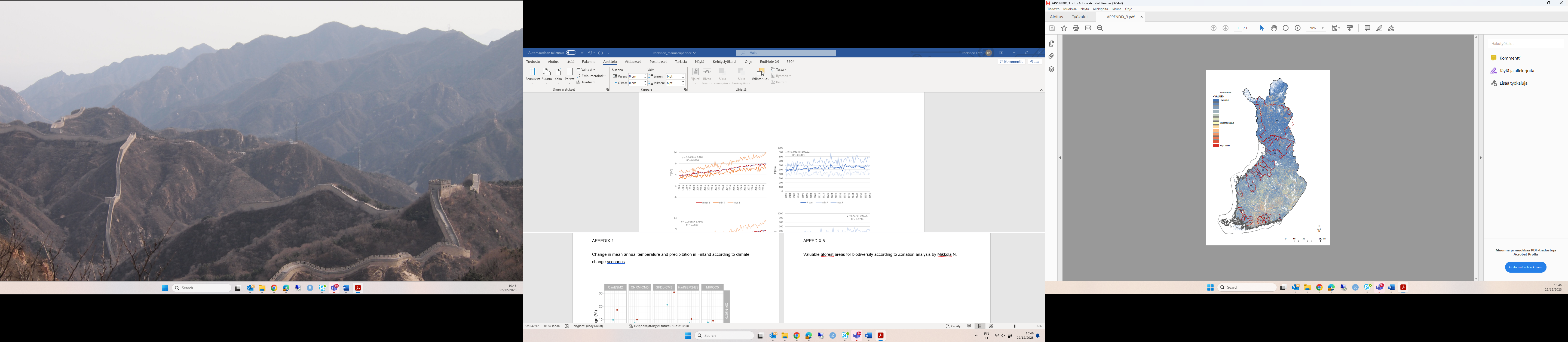

Supplement: Supplementary file 1 — Supplementary information [file 267_2024_2058_MOESM1_ESM.docx]
